# Supplementary material for: CpG ODN D35 improves the response to abbreviated low-dose pentavalent antimonial treatment in non-human primate model of cutaneous leishmaniasis
Source: PLoS Negl Trop Dis. 2020 Feb 28;14(2):e0008050. doi: 10.1371/journal.pntd.0008050 (PMC7075640; doi:10.1371/journal.pntd.0008050)
Supplement: S1 Methods — (DOCX) [file pntd.0008050.s001.docx]

Supporting Information

**S1 Fig. Design of dose escalation study in Cynomolgus Macaques**. Cynomolgus monkeys were injected SC on the chest with increasing doses of D35 every 3 weeks. Blood was collected immediately preceding each administration of D35. Skin biopsies were performed 24 hours after D35 treatment. Blood was collected 1 day and 1 week post-treatment. The animals were rested for 2 weeks between D35 treatments. A final cycle where the macaques received saline was performed to determine whether the gene expression levels had returned to baseline.

**S2 Fig. Graphical representation of *L. major* infection and treatment study**. Rhesus macaques were infected ID on the forehead in three separate location with *L. major*. Lesion size was monitored every 2-3 days until more than 70% of the animals had a lesion greater than 3mm^2^. Animals were divided into the four groups diagrammed above. Lesion size and treatments were administered as indicated above.

**S3 Fig. Physical characterization of ultrapure and research grade D35.** Ultrapure D35 shows reduced process related impurities by RP- HPLC (A), IEX-HPLC (B), gel electrophoresis (C) and micro flow imaging (D) upon reconstitution. A RP-HPLC (XBridge 4.6×75 mm column, mobile phase A 8 mM TEA/100 mM HFIP, mobile phase B MeOH, gradient B 5→30％ in 20 min., flow rate 1 mL/min., column temperature 60°C and wavelength 260 nm). Samples were resuspended in water for injection and filtered with 0.22um membrane. Four ul of sample was injected on a Water Alliance system with a Xbridge 4.6x74mm column.; B IEX-HPLC (DNAPac^TM^ PA200, mobile phase A 20 mM Tris-HCl pH 7.5/20% acetonitrile, mobile phase B 400 mM NaClO_4_/20 mM Tris-HCl pH 7.5/20% acetonitrile, gradient B 0→100％ in 30 min., flow rate 1mL/min., column temperature 60°C and wavelength 260 nm). Samples were resuspended in water for injection and filtered with 0.22um membrane. Four ul of sample was injected on a Shimadzu Prominence system with a DNAPac^TM^ PA200 column*.* (C) ODNs (0.3–1 OD260) were analyzed by gel electrophoresis under non-denaturing conditions (20% polyacrylamide and 1× TBE, pH 8.3) at ∼25°C. The gel was electrophoresed at 250 V until the bromophenol blue marker (fastest moving band marked with black triangle) had traveled 20 cm and then stained with Stains-all. Electrophoretic mobility of monomeric ODN is indicated by a solid arrow, whereas the open arrow shows the mobility of ODN exhibiting secondary structures and/or self-complementary dimerization. Note that research D35 ODN has higher order structures that are absent from the ultrapure D35. (D) Microflow imaging. Following reconstitution research and ultrapure CpG ODNs were diluted to 100ug ODN/ml in 0.1uM filtered water and the number of particles counted.

**S4 Fig. Low dose Sb^V^ results in recrudescence.** Macaques were infected with *L. major* and were treated with saline, High dose Sb^V^ (20mg/kg 20days), or low dose Sb^V^ (5mg/kg for 5 days). Lesions were measured at least weekly. A) All animals treated with high dose SbV showed complete healing. B) Macaques treated with low dose Sb^V^ showed slower healing time and recrudescence in a subset of animals.

**S5 Fig. Evolution of *L. major* lesions.**

Images of lesions of each of the animals at Day8 (preD35), D11 (3 days post D35), and D36. A) Graphical schematic of the study design. Animals were infected on day 1, and their lesions evaluated every 2-3 days. On day 8, after 14 animals had lesions, 7 animals were treated with CpG ODN (1 mg/kg SC) or saline. Three days later the 8 macaques were started on Sb^V^ (5 mg/kg/day IM) for 10 days. The lesions sizes were evaluated every 5-7 days after the start of treatment until week 10 when most lesions had resolved. B) images for each animal taken at the indicated time point. Border color of each images indicates which treatment the macaque received.

**S6 Fig. Detection of gene signature in PMBCs of macaques treated with D35.**

A distinct signature of D35 treatment was easily detected in circulation of macaques 3 days post D35 administration in *L. major* infected animals. (A) Magnitude of the D35 response in PB was assessed by calculated a gene score of the top eight type I IFN induced genes, and D35 animals displayed a significant increase compared to saline injected animals. No difference could be detected at the lesion site. Genes used to calculate the gene score were IFIT1, 2, 3, MX1, OAS2, IRF7, CCL2, and 22. B) Gene induction observed by qPCR was comparable to that determined by nanostring analysis. Saline group contained 7 animals and the D35 group contained 8 animals. P < 0.01 indicated by **.

**S7 Fig. Gene signature in skin of macaques treated with D35.**

Heat map shows the mean of the shared genes altered in *L. major* infected saline and D35 treated animals. Clustering was generated using Ward’s minimum variance method in R. p <0.001 indicated by *** was calculated by ANOVA

**S8 Fig. Leishmania quantification by minicircle qPCR**.

Numbers of *L. major* parasites were quantified by qPCR using minicircle DNA isolated from a portion of a skin biopsy. Using two different primers targeting separate sequences. Absolute numbers of parasites were calculated using a standard curve generated from biopsy samples spiked with a known number of parasites or the relative quantity of parasites were calculated by standardizing to the rhGapdh. Parasites numbers were quantified at D11, D22 and D71 post infection.
