# Supplementary figures and images for: CpG ODN D35 improves the response to abbreviated low-dose pentavalent antimonial treatment in non-human primate model of cutaneous leishmaniasis
Source: PLoS Negl Trop Dis. 2020 Feb 28;14(2):e0008050. doi: 10.1371/journal.pntd.0008050 (PMC7075640; doi:10.1371/journal.pntd.0008050)

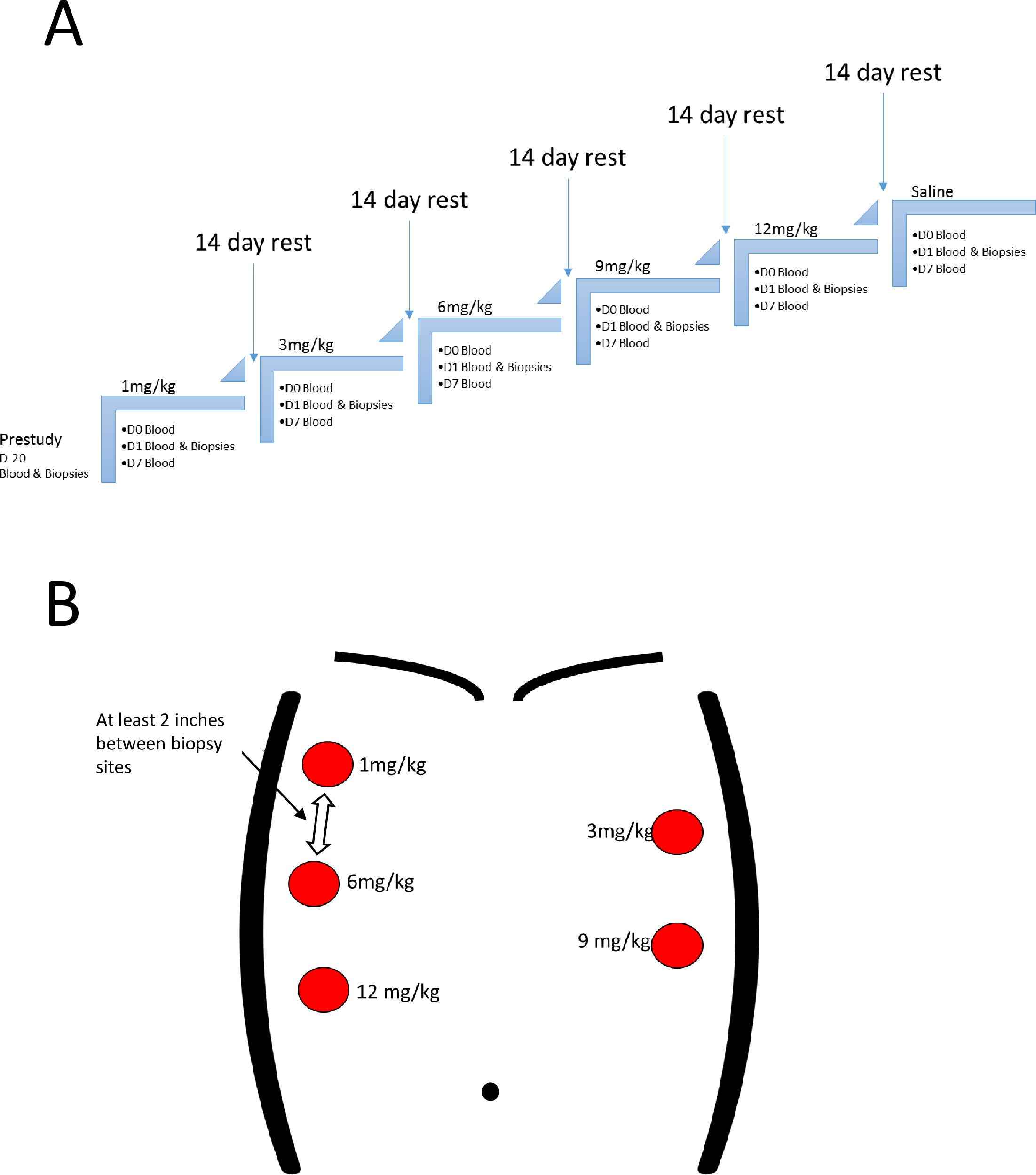

Supplement: S1 Fig — (TIF) [file pntd.0008050.s002.tif]

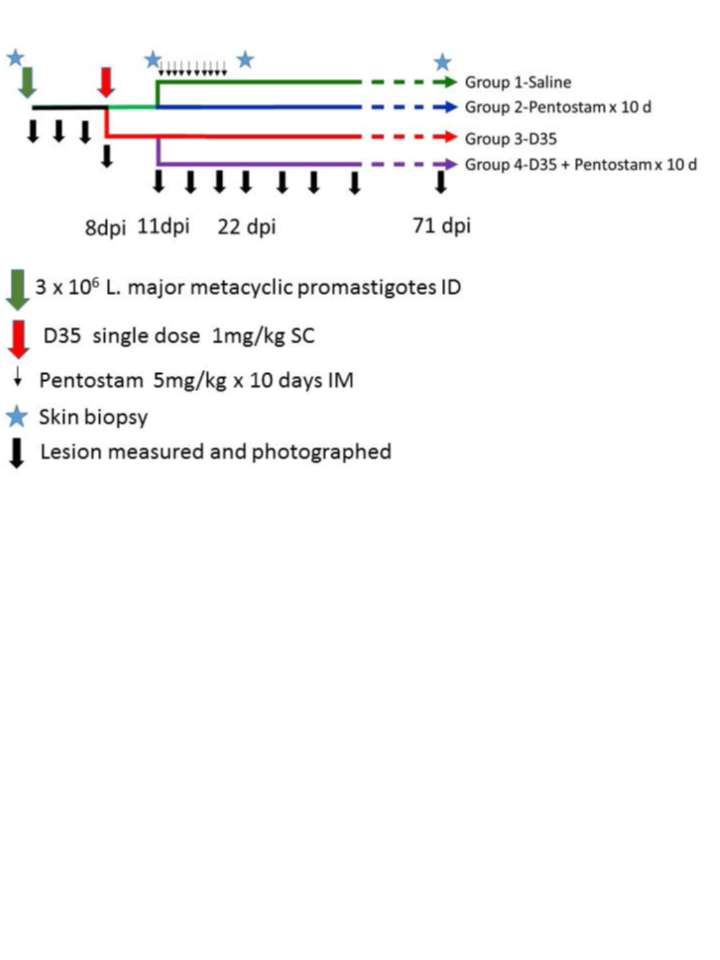

Supplement: S2 Fig — (TIF) [file pntd.0008050.s003.tif]

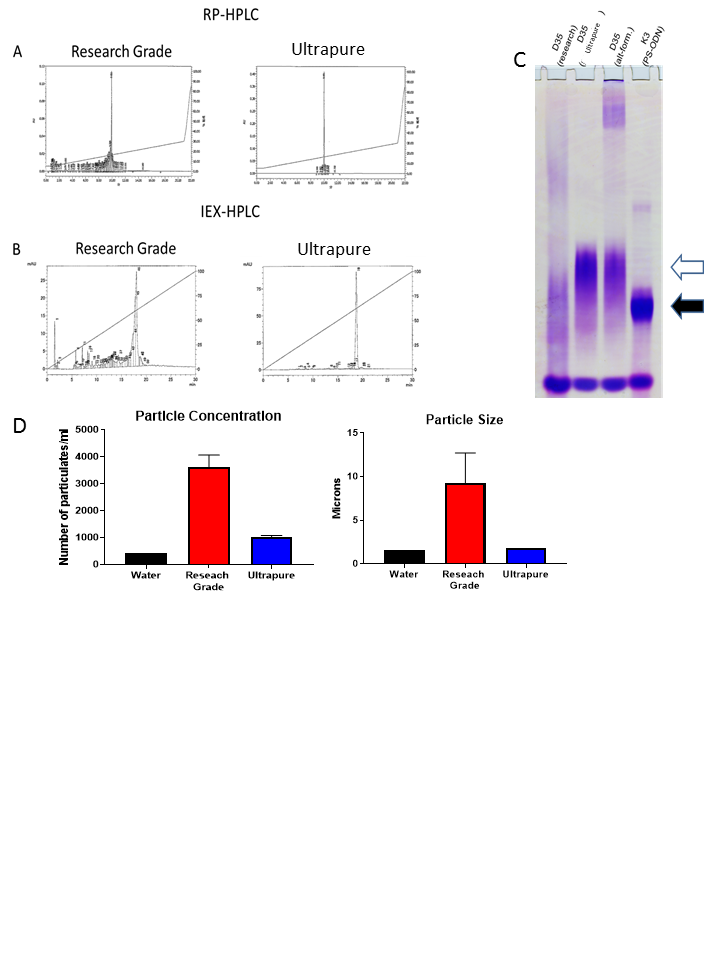

Supplement: S3 Fig — (TIF) [file pntd.0008050.s004.tif]

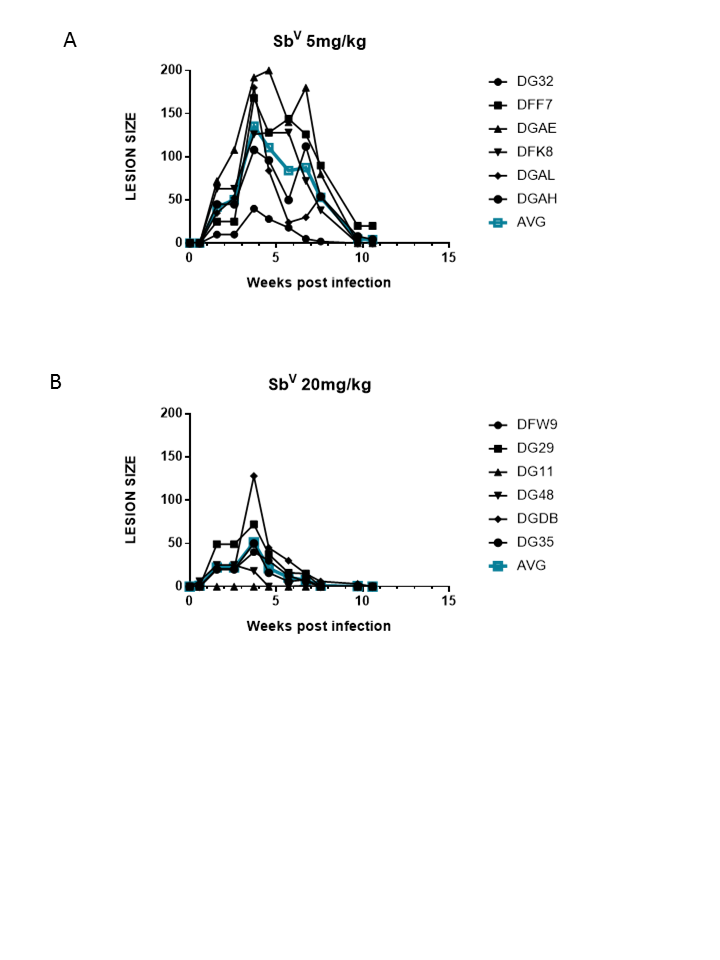

Supplement: S4 Fig — (TIF) [file pntd.0008050.s005.tif]

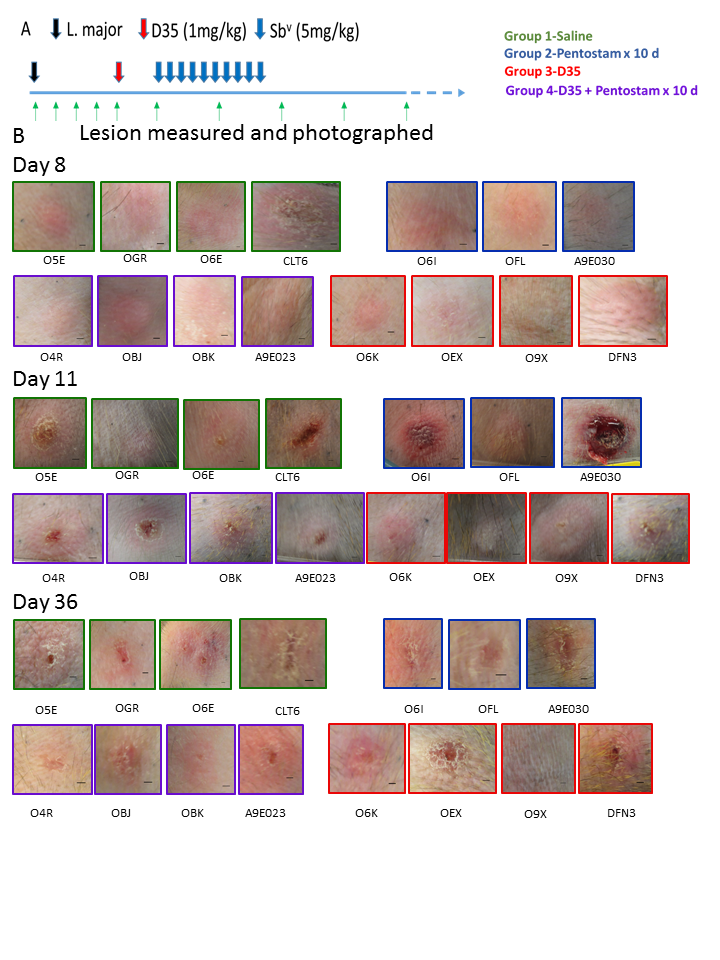

Supplement: S5 Fig — (TIF) [file pntd.0008050.s006.tif]

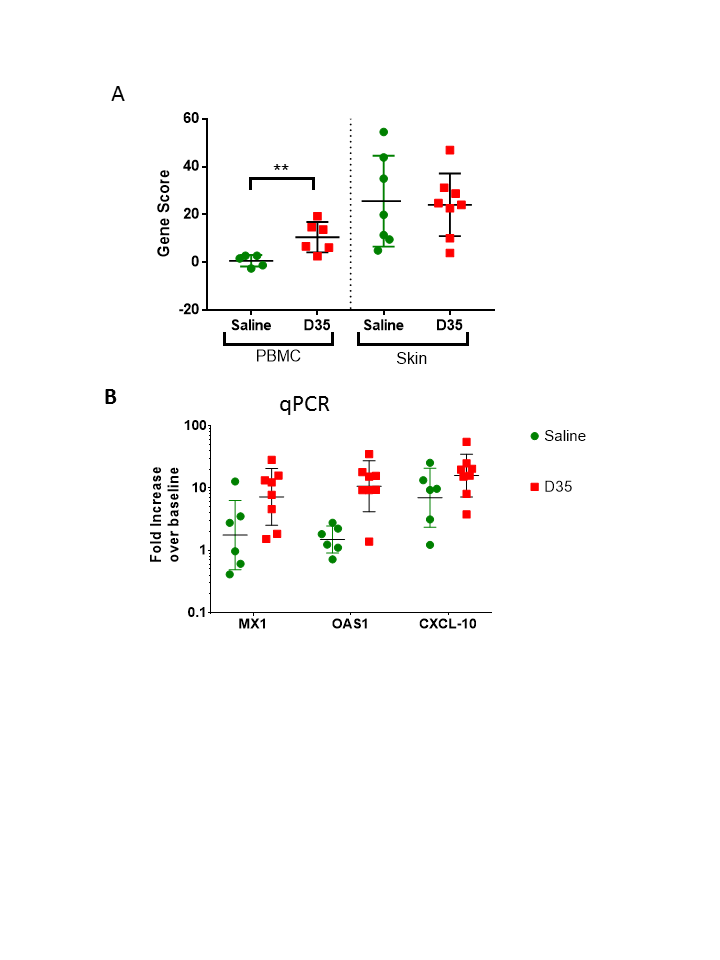

Supplement: S6 Fig — (TIF) [file pntd.0008050.s007.tif]

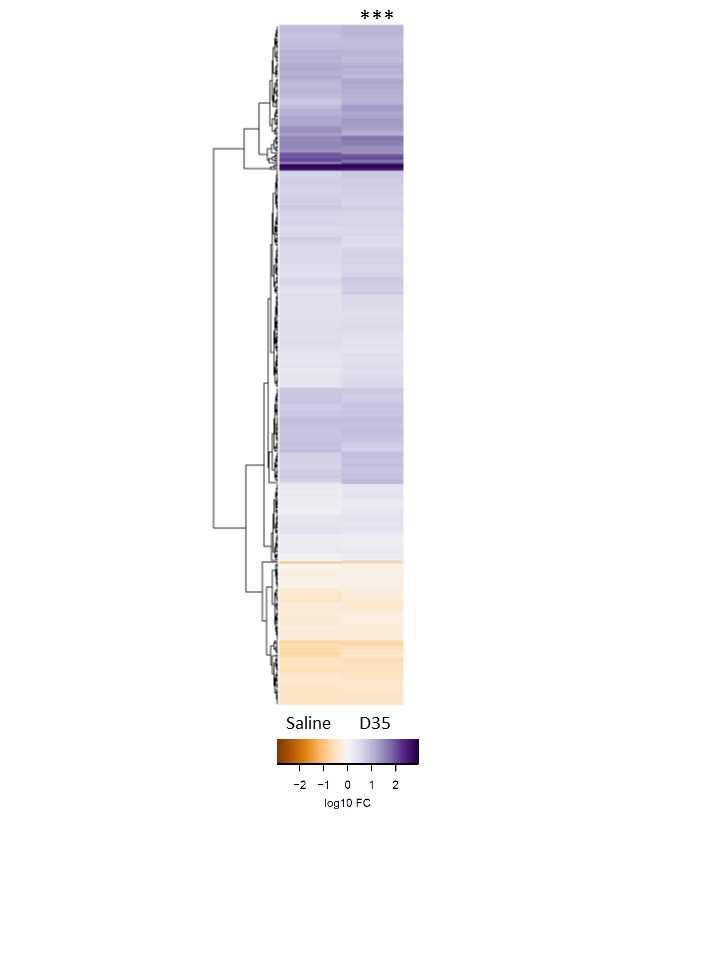

Supplement: S7 Fig — (TIF) [file pntd.0008050.s008.tif]

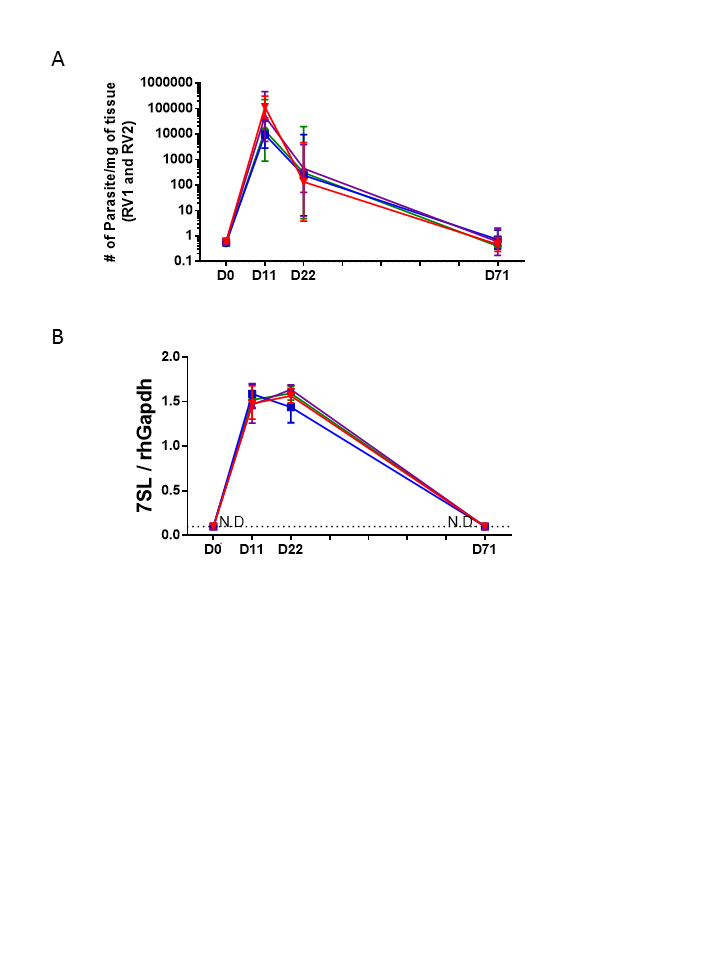

Supplement: S8 Fig — (TIF) [file pntd.0008050.s009.tif]
