# Supplementary material for: CpG ODN D35 improves the response to abbreviated low-dose pentavalent antimonial treatment in non-human primate model of cutaneous leishmaniasis
Source: PLoS Negl Trop Dis. 2020 Feb 28;14(2):e0008050. doi: 10.1371/journal.pntd.0008050 (PMC7075640; doi:10.1371/journal.pntd.0008050)
Supplement: S1 Table — (DOCX) [file pntd.0008050.s010.docx]

| Supplementary Table I. Genes in Custom NanoString code set. |
| --- |
| Gene ID |
| ARG1 |
| BCL2 |
| C3 |
| CASP1 |
| CASP8 |
| CCL17 |
| CCL2 |
| CCL20 |
| CCL22 |
| CCL3 |
| CCL5 |
| CCL8 |
| CCR2 |
| CCR5 |
| CCR6 |
| CCR7 |
| CD14 |
| CD163 |
| CD209 |
| CD3E |
| CD4 |
| CD40 |
| CD40LG |
| CD80 |
| CD86 |
| CD8A |
| CXCL10 |
| FOXP3 |
| GNLY |
| GUSB |
| GZMB |
| HPRT1 |
| IDO1 |
| IFIT1 |
| IFIT2 |
| IFIT3 |
| IFNA1 |
| IFNA2 |
| IFNAR1 |
| IFNB1 |
| IFNG |
| IL10 |
| IL10RA |
| IL10RB |
| IL12A |
| IL12B |
| IL12RB1 |
| IL13 |
| IL15 |
| IL17A |
| IL17F |
| IL17RA |
| IL1A |
| IL1B |
| IL20 |
| IL22 |
| IL23A |
| IL6 |
| IL8 |
| IRF3 |
| IRF7 |
| KIR2DL4 |
| MMP9 |
| MX1 |
| NCR1 |
| NOS2 |
| OAS2 |
| OAZ1 |
| POLR2A |
| PRF1 |
| S100A8 |
| S100A9 |
| SDHA |
| SELE |
| SOCS1 |
| SOCS3 |
| STAT1 |
| STAT3 |
| TBP |
| TBX21 |
| TGFB1 |
| TLR7 |
| TLR9 |
| TNF |
| XCL1 |
|  |
