# Supplementary material for: CpG ODN D35 improves the response to abbreviated low-dose pentavalent antimonial treatment in non-human primate model of cutaneous leishmaniasis
Source: PLoS Negl Trop Dis. 2020 Feb 28;14(2):e0008050. doi: 10.1371/journal.pntd.0008050 (PMC7075640; doi:10.1371/journal.pntd.0008050)
Supplement: S2 Table — (DOCX) [file pntd.0008050.s011.docx]

| **Supplementary Table II. Ingenuity Canonical Pathways.** |
| --- |

| Pathway name | -log(p-value) | Ratio | z-score |
| --- | --- | --- | --- |
| TREM1 Signaling | 29.6 | 0.28 | 4.583 |
| Neuroinflammation Signaling Pathway | 27.6 | 0.0958 | 5.014 |
| Altered T Cell and B Cell Signaling in Rheumatoid Arthritis | 24.2 | 0.211 |  |
| Crosstalk between Dendritic Cells and Natural Killer Cells | 20.9 | 0.191 |  |
| Th1 and Th2 Activation Pathway | 20.7 | 0.112 |  |
| Dendritic Cell Maturation | 20.3 | 0.107 | 4.359 |
| Role of Macrophages, Fibroblasts and Endothelial Cells in Rheumatoid Arthritis | 19.4 | 0.0743 |  |
| Hepatic Fibrosis / Hepatic Stellate Cell Activation | 19.3 | 0.108 |  |
| Type I Diabetes Mellitus Signaling | 19.1 | 0.153 | 3.051 |
| Toll-like Receptor Signaling | 18.7 | 0.197 | 2.111 |
| Communication between Innate and Adaptive Immune Cells | 18.7 | 0.167 |  |
| Role of Pattern Recognition Receptors in Recognition of Bacteria and Viruses | 17.4 | 0.122 | 3.464 |
| T Helper Cell Differentiation | 17.3 | 0.192 |  |
| Granulocyte Adhesion and Diapedesis | 16.8 | 0.1 |  |
| Th1 Pathway | 16.1 | 0.117 | 2.53 |
| Graft-versus-Host Disease Signaling | 14.6 | 0.229 |  |
| IL-10 Signaling | 14.3 | 0.174 |  |
| Th2 Pathway | 14 | 0.0987 | 2.333 |
| Agranulocyte Adhesion and Diapedesis | 13.7 | 0.0833 |  |
| LXR/RXR Activation | 12.7 | 0.107 | -3.051 |
| Atherosclerosis Signaling | 12.3 | 0.102 |  |
| Role of Hypercytokinemia/hyperchemokinemia in the Pathogenesis of Influenza | 11.6 | 0.209 |  |
| T Cell Exhaustion Signaling Pathway | 11.6 | 0.0769 | 2.309 |
| Th17 Activation Pathway | 11.4 | 0.121 | 3.317 |
| NF-κB Signaling | 11.4 | 0.0749 | 2.673 |
| Hepatic Cholestasis | 11.1 | 0.0812 |  |
| HMGB1 Signaling | 10.5 | 0.0851 | 3.317 |
| Differential Regulation of Cytokine Production in Intestinal Epithelial Cells by IL-17A and IL-17F | 10.4 | 0.304 |  |
| Allograft Rejection Signaling | 10.2 | 0.116 |  |
| iNOS Signaling | 9.78 | 0.178 | 2.828 |
| STAT3 Pathway | 9.58 | 0.0833 | -0.447 |
| Phagosome Formation | 9.58 | 0.0833 |  |
| Differential Regulation of Cytokine Production in  Macrophages and T Helper Cells by IL-17A and IL-17F | 9.26 | 0.333 |  |
| Glucocorticoid Receptor Signaling | 8.84 | 0.0432 |  |
| IL-6 Signaling | 8.22 | 0.0735 | 2.333 |
| Role of IL-17A in Arthritis | 8.19 | 0.114 |  |
| FAT10 Cancer Signaling Pathway | 8.12 | 0.152 | 2.646 |
| Autoimmune Thyroid Disease Signaling | 7.92 | 0.143 |  |
| TNFR2 Signaling | 7.78 | 0.2 | 2 |
| Production of Nitric Oxide and Reactive Oxygen Species in Macrophages | 7.76 | 0.0561 | 3.162 |
| Osteoarthritis Pathway | 7.43 | 0.0521 | 3.162 |
| IL-17A Signaling in Fibroblasts | 7.36 | 0.171 |  |
| Induction of Apoptosis by HIV1 | 7.24 | 0.115 | 1.89 |
| Complement System | 7.2 | 0.162 |  |
| Acute Phase Response Signaling | 7.15 | 0.0568 | 2.333 |
| Pathogenesis of Multiple Sclerosis | 6.9 | 0.444 |  |
| Role of IL-17F in Allergic Inflammatory Airway Diseases | 6.68 | 0.133 | 2.236 |
| Role of JAK1, JAK2 and TYK2 in Interferon Signaling | 6.66 | 0.208 |  |
| Colorectal Cancer Metastasis Signaling | 6.6 | 0.0431 | 3.317 |
| CD40 Signaling | 6.38 | 0.0864 | 0.816 |
| iCOS-iCOSL Signaling in T Helper Cells | 6.21 | 0.064 | 2.236 |
| OX40 Signaling Pathway | 6.07 | 0.0778 | 1 |
| Role of Osteoblasts, Osteoclasts and Chondrocytes in Rheumatoid Arthritis | 5.99 | 0.0426 |  |
| CD28 Signaling in T Helper Cells | 5.98 | 0.0597 | 1 |
| Systemic Lupus Erythematosus Signaling | 5.97 | 0.0424 |  |
| MIF-mediated Glucocorticoid Regulation | 5.86 | 0.147 | 2.236 |
| Activation of IRF by Cytosolic Pattern Recognition Receptors | 5.79 | 0.0952 | 1.633 |
| Role of NFAT in Regulation of the Immune Response | 5.76 | 0.0466 | 2.828 |
| B Cell Development | 5.73 | 0.139 |  |
| Interferon Signaling | 5.73 | 0.139 | 2.236 |
| PPAR Signaling | 5.73 | 0.0693 | -2.646 |
| Antigen Presentation Pathway | 5.61 | 0.132 |  |
| Role of PKR in Interferon Induction and Antiviral Response | 5.44 | 0.122 | #NUM! |
| MIF Regulation of Innate Immunity | 5.39 | 0.119 | 2.236 |
| MSP-RON Signaling Pathway | 5.38 | 0.0811 | #NUM! |
| Inflammasome pathway | 5.34 | 0.2 | 2 |
| Chemokine Signaling | 5.28 | 0.0779 | 2.449 |
| IL-17A Signaling in Airway Cells | 5.18 | 0.075 | 1.342 |
| IL-9 Signaling | 5.15 | 0.106 | 0 |
| Airway Pathology in Chronic Obstructive Pulmonary Disease | 5 | 0.375 |  |
| Natural Killer Cell Signaling | 5 | 0.0538 |  |
| Small Cell Lung Cancer Signaling | 4.97 | 0.069 | 2.236 |
| JAK/Stat Signaling | 4.86 | 0.0659 | -0.816 |
| Role of Cytokines in Mediating Communication between Immune Cells | 4.84 | 0.0926 |  |
| IL-17 Signaling | 4.8 | 0.0645 |  |
| Death Receptor Signaling | 4.8 | 0.0645 | 1.633 |
| Apoptosis Signaling | 4.72 | 0.0625 | 1.633 |
| Nur77 Signaling in T Lymphocytes | 4.66 | 0.0847 |  |
| IL-12 Signaling and Production in Macrophages | 4.63 | 0.0473 |  |
| LPS/IL-1 Mediated Inhibition of RXR Function | 4.36 | 0.0359 | 2.828 |
| TWEAK Signaling | 4.33 | 0.114 | 0 |
| Lymphotoxin β Receptor Signaling | 4.32 | 0.0725 | 1 |
| Role of IL-17A in Psoriasis | 4.3 | 0.231 |  |
| PKCθ Signaling in T Lymphocytes | 4.3 | 0.0419 | 2.646 |
| p38 MAPK Signaling | 4.22 | 0.0508 | 2.449 |
| April Mediated Signaling | 4.14 | 0.103 | 1 |
| Role of JAK1 and JAK3 in γc Cytokine Signaling | 4.07 | 0.0641 |  |
| B Cell Activating Factor Signaling | 4.06 | 0.0976 |  |
| Role of MAPK Signaling in the Pathogenesis of Influenza | 4.04 | 0.0633 |  |
| Role of PI3K/AKT Signaling in the Pathogenesis of Influenza | 4.02 | 0.0625 | 1.342 |
| Role of RIG1-like Receptors in Antiviral Innate Immunity | 3.94 | 0.0909 |  |
| IL-15 Signaling | 3.91 | 0.0595 |  |
| B Cell Receptor Signaling | 3.89 | 0.0361 | 0.816 |
| Aryl Hydrocarbon Receptor Signaling | 3.77 | 0.0423 | 0.816 |
| IL-8 Signaling | 3.76 | 0.0343 | 2.646 |
| TNFR1 Signaling | 3.72 | 0.08 |  |
| CCR5 Signaling in Macrophages | 3.68 | 0.0532 |  |
| Sumoylation Pathway | 3.64 | 0.0521 |  |
| Leukocyte Extravasation Signaling | 3.64 | 0.0329 | 2.449 |
| Tumoricidal Function of Hepatic Natural Killer Cells | 3.47 | 0.125 |  |
| IL-17A Signaling in Gastric Cells | 3.42 | 0.12 |  |
| Neuroprotective Role of THOP1 in Alzheimer's Disease | 3.2 | 0.0417 | 1.342 |
| PPARα/RXRα Activation | 3.15 | 0.0323 | -1.342 |
| Cytotoxic T Lymphocyte-mediated Apoptosis of Target Cells | 3.1 | 0.0938 |  |
| 4-1BB Signaling in T Lymphocytes | 3.1 | 0.0938 |  |
| Renin-Angiotensin Signaling | 3.04 | 0.0385 | 2.236 |
| VDR/RXR Activation | 2.98 | 0.0513 | 2 |
| PI3K Signaling in B Lymphocytes | 2.97 | 0.037 | 2.236 |
| Erythropoietin Signaling | 2.79 | 0.0455 |  |
| IL-1 Signaling | 2.73 | 0.044 | 2 |
| Type II Diabetes Mellitus Signaling | 2.7 | 0.0323 | 2 |
| LPS-stimulated MAPK Signaling | 2.66 | 0.0421 | 2 |
| NF-κB Activation by Viruses | 2.66 | 0.0421 |  |
| IL-4 Signaling | 2.65 | 0.0417 |  |
| Acute Myeloid Leukemia Signaling | 2.6 | 0.0404 | 0 |
| CTLA4 Signaling in Cytotoxic T Lymphocytes | 2.57 | 0.0396 |  |
| Hematopoiesis from Pluripotent Stem Cells | 2.56 | 0.0612 |  |
| Tec Kinase Signaling | 2.52 | 0.0292 |  |
| HGF Signaling | 2.29 | 0.0331 |  |
| Pancreatic Adenocarcinoma Signaling | 2.27 | 0.0328 | 2 |
| ILK Signaling | 2.26 | 0.0254 | 2 |
| 3-phosphoinositide Biosynthesis | 2.21 | 0.0248 | 2.236 |
| Calcium-induced T Lymphocyte Apoptosis | 2.19 | 0.0455 |  |
| PI3K/AKT Signaling | 2.19 | 0.031 | 1 |
| Role of JAK family kinases in IL-6-type Cytokine Signaling | 2.05 | 0.08 |  |
| Glioma Invasiveness Signaling | 1.99 | 0.0385 |  |
| IL-15 Production | 1.95 | 0.0714 |  |
| Superpathway of Inositol Phosphate Compounds | 1.93 | 0.0211 | 2.236 |
| Regulation of IL-2 Expression in Activated and Anergic T Lymphocytes | 1.88 | 0.0349 |  |
| Macropinocytosis Signaling | 1.84 | 0.0337 |  |
| Cdc42 Signaling | 1.81 | 0.024 |  |
| Role of JAK2 in Hormone-like Cytokine Signaling | 1.79 | 0.0588 |  |
| Coagulation System | 1.77 | 0.0571 |  |
| Bladder Cancer Signaling | 1.77 | 0.0319 |  |
| PEDF Signaling | 1.76 | 0.0316 |  |
| Ceramide Signaling | 1.69 | 0.0297 |  |
| Inhibition of Matrix Metalloproteases | 1.68 | 0.0513 |  |
| Prostate Cancer Signaling | 1.67 | 0.0291 |  |
| Oncostatin M Signaling | 1.66 | 0.05 |  |
| Airway Inflammation in Asthma | 1.64 | 0.25 |  |
| Cholecystokinin/Gastrin-mediated Signaling | 1.63 | 0.028 |  |
| Antioxidant Action of Vitamin C | 1.61 | 0.0275 |  |
| Telomerase Signaling | 1.51 | 0.0252 |  |
| Primary Immunodeficiency Signaling | 1.48 | 0.04 |  |
| PTEN Signaling | 1.47 | 0.0244 |  |
| FXR/RXR Activation | 1.45 | 0.0238 |  |
| Apelin Endothelial Signaling Pathway | 1.44 | 0.0236 |  |
| CD27 Signaling in Lymphocytes | 1.43 | 0.0377 |  |
| fMLP Signaling in Neutrophils | 1.42 | 0.0233 |  |
|  |  |  |  |
