# Supplementary material for: CpG ODN D35 improves the response to abbreviated low-dose pentavalent antimonial treatment in non-human primate model of cutaneous leishmaniasis
Source: PLoS Negl Trop Dis. 2020 Feb 28;14(2):e0008050. doi: 10.1371/journal.pntd.0008050 (PMC7075640; doi:10.1371/journal.pntd.0008050)
Supplement: S3 Table — (DOCX) [file pntd.0008050.s012.docx]

| **Supplementary Table III. Clinical chemistry values and temperatures for cynomolgus macaques** | | | | | | | | | | |
| --- | --- | --- | --- | --- | --- | --- | --- | --- | --- | --- |
|  | | 1mg/kg D35 | | | 3mg/kg D35 | | | 6mg/kg D35 | | |
|  |  | Pre Admin | 24 hour Post Admin | 7 Days Post Admin | Pre Admin | 24 hour Post Admin | 7 Days Post Admin | Pre Admin | 24 hour Post Admin | 7 Days Post Admin |
|  |  | Mean (SD) | Mean (SD) | Mean (SD) | Mean (SD) | Mean (SD) | Mean (SD) | Mean (SD) | Mean (SD) | Mean (SD) |
| Sodium | mmol/L | 145.7 (2.3) | 145.7 (1.2) | 147.7 (0.6) | 147.7 (1.5) | 145 (3.5) | 146.7 (2.1) | 146.7 (1.5) | 146 (2.6) | 146.3 (1.5) |
| Potassium | mmol/L | 4.4 (0.3) | 6.4 (1) | 4.5 (0.4) | 4.3 (0.3) | 6.6 (2.1) | 4.2 (0) | 4.5 (0.5) | 4.2 (0.2) | 4.7 (0.8) |
| Chloride | mmol/L | 108.7 (2.3) | 105.7 (1.5) | 110 (1) | 110.3 (1.5) | 106.7 (2.9) | 105.7 (2.5) | 108 (1.7) | 105.7 (4.5) | 106.7 (0.6) |
| Calcium | mmol/L | 2.4 (0.1) | 2.3 (0.1) | 2.4 (0.2) | 2.4 (0.1) | 2.3 (0) | 2.3 (0.1) | 2.3 (0.1) | 2.4 (0.1) | 2.4 (0.2) |
| Magnesium | mmol/L | 0.9 (0.1) | 1 (0.1) | 0.8 (0) | 0.9 (0.1) | 0.8 (0.1) | 0.8 (0) | 0.9 (0) | 0.8 (0) | 0.8 (0.1) |
| Inorganic Phosphorus | mg/dL | 4.8 (0.6) | 5.4 (0.6) | 4 (1) | 4 (0.5) | 4.6 (1.2) | 4.1 (1.8) | 4 (1.1) | 4.9 (1.6) | 4.2 (2) |
| Glucose | mg/dL | 71.3 (11.5) | 73 (10.4) | 70.3 (9) | 74.3 (7.6) | 65 (11) | 80 (7.2) | 70 (19.9) | 89.3 (19.9) | 79 (13.2) |
| BUN | mg/dL | 19 (6.2) | 24.7 (5.5) | 20 (7) | 22.3 (10.1) | 22.3 (9.7) | 18.7 (4.7) | 29.3 (18.9) | 18.3 (5.8) | 19.3 (4) |
| Creatine | mg/dL | 0.8 (0.1) | 0.9 (0.1) | 0.8 (0.2) | 0.7 (0.2) | 0.9 (0.1) | 0.7 (0.1) | 0.8 (0.1) | 0.9 (0.1) | 0.7 (0.1) |
| Uric Acid | mg/dL | 0.2 (0) | 0.2 (0) | 0.2 (0) | 0.2 (0) | 0.2 (0) | 0.2 (0) | 0.2 (0) | 0.2 (0) | 0.2 (0) |
| Albumin | g/dL | 3.9 (0.1) | 3.8 (0.1) | 3.8 (0.2) | 3.9 (0) | 4 (0.1) | 3.8 (0.1) | 3.9 (0.2) | 3.9 (0.2) | 3.8 (0.2) |
| Total Protein | g/dL | 6.9 (0.2) | 6.9 (0.2) | 6.6 (0.4) | 6.8 (0.2) | 6.9 (0) | 6.7 (0.2) | 6.9 (0.4) | 6.7 (0.3) | 6.8 (0.2) |
| Cholesterol | mg/dL | 98 (9.8) | 90.3 (11.8) | 87.3 (18.8) | 89.7 (24.7) | 93.3 (34.6) | 91.7 (11.5) | 85.3 (16.9) | 81.7 (16.9) | 93 (12.5) |
| Triglycerides | mg/dL | 72 (58.9) | 86.3 (58.9) | 56.3 (44.7) | 84.3 (37.8) | 106.3 (58.4) | 58 (33.3) | 99.7 (29.1) | 85.3 (36.6) | 71.3 (38.2) |
| Alkaline Phosphatase | U/L | 123.3 (30.4) | 120.7 (23.2) | 105 (26.6) | 118 (39.9) | 119.3 (39.5) | 110 (43.3) | 111.7 (41.4) | 111.7 (39.6) | 99 (33.6) |
| ALT/GPT | U/L | 36.3 (6.1) | 39.7 (10.7) | 37.3 (8.4) | 41.3 (13.1) | 50 (11) | 41 (8.7) | 44.7 (15.5) | 46.3 (13.3) | 43.3 (10.5) |
| AST/GOT | U/L | 33.3 (6.7) | 66 (41.1) | 25 (2.6) | 33.3 (9) | 75 (28) | 35.7 (11.6) | 48.7 (7.6) | 54.3 (8.5) | 46.7 (3.1) |
| Amylase | U/L | 341.7 (102.5) | 389.3 (108.3) | 339.7 (124.6) | 357.7 (144.2) | 366.7 (86.7) | 346.3 (119) | 365 (126) | 363.7 (122.4) | 363.7 (118) |
| Total CK | U/L | 1723.7 (2193.6) | 2637.7 (3004.4) | 583.7 (156) | 474 (296.2) | 2029.3 (387.4) | 402.7 (220) | 616.3 (226.1) | 1481 (716.8) | 356 (116.6) |
| LD | U/L | 400.3 (34.8) | 1523.3 (467.6) | 309.3 (59.7) | 584 (609.3) | 1131.7 (186.6) | 829.7 (586.2) | 972 (360.4) | 805.3 (436.8) | 916 (179.5) |
| Total Bilirubin | mg/dL | 0.2 (0) | 0.2 (0) | 0.2 (0) | 0.2 (0) | 0.2 (0) | 0.2 (0) | 0.2 (0) | 0.2 (0) | 0.2 (0) |
| Direct Bilirubin | mg/dL | 0.2 (0) | 0.2 (0) | 0.2 (0) | 0.2 (0) | 0.2 (0) | 0.2 (0) | 0.2 (0) | 0.2 (0) | 0.2 (0) |
| Temperature | C |  | 100.6(1.1) |  | 99.9(0.7) | 100.2(0.2) |  | 100.1(0.6) | 100.4(0.2) |  |

| Supplementary Table III. Clinical chemistry values and temperature for Cynomolgus Macaques continued | | | | | | | | | | |
| --- | --- | --- | --- | --- | --- | --- | --- | --- | --- | --- |
|  | | 9mg/kg D35 | | | 12mg/kg D35 | | | 0mg/kg D35 | | |
|  |  | Pre Admin | 24 hour Post Admin | 7 Days Post Admin | Pre Admin | 24 hour Post Admin | 7 Days Post Admin | Pre Admin | 24 hour Post Admin | 7 Days Post Admin |
|  |  | Mean (SD) | Mean (SD) | Mean (SD) | Mean (SD) | Mean (SD) | Mean (SD) | Mean (SD) | Mean (SD) | Mean (SD) |
| Sodium | mmol/L | 146.7 (1.5) | 145.3 (3.8) | 148 (1.7) | 146 (2) | 145.7 (1.5) | 146.8 (3.1) | 147.3 (0.6) | 146.3 (3.5) | 148.3 (1.2) |
| Potassium | mmol/L | 4.6 (0.1) | 4.9 (0.8) | 4.3 (0.2) | 4.1 (0.2) | 5.5 (0.8) | 5.3 (0.6) | 4.4 (0.3) | 4.3 (0.2) | 4.3 (0.2) |
| Chloride | mmol/L | 107.3 (1.5) | 104 (3.5) | 108.3 (2.1) | 104.7 (2.5) | 105 (1.7) | 107.3 (1.9) | 107.7 (0.6) | 105.7 (2.5) | 108.7 (1.2) |
| Calcium | mmol/L | 2.4 (0.1) | 2.4 (0.1) | 2.3 (0.1) | 2.4 (0.1) | 2.3 (0) | 2.3 (0.1) | 2.4 (0.1) | 2.5 (0.1) | 2.4 (0) |
| Magnesium | mmol/L | 0.9 (0) | 0.8 (0.1) | 0.8 (0) | 0.8 (0) | 0.8 (0.1) | 0.8 (0) | 0.8 (0.1) | 0.8 (0.1) | 0.8 (0.1) |
| Inorganic Phosphorus | mg/dL | 3.5 (0.4) | 4.2 (0.6) | 3.8 (1.4) | 4.9 (0.7) | 4.9 (1.5) | 4.5 (1.9) | 4.8 (2) | 4.7 (0.6) | 4.9 (1.3) |
| Glucose | mg/dL | 69 (6.1) | 80.7 (2.1) | 67.7 (2.1) | 75 (10.8) | 80 (11.4) | 75.3 (2.5) | 73.3 (12.1) | 78 (12) | 67.7 (14.6) |
| BUN | mg/dL | 20.7 (0.6) | 22 (5.6) | 19 (1.7) | 18.3 (1.5) | 21 (2) | 20.8 (3.9) | 19.7 (3.5) | 20.7 (0.6) | 19.3 (4.5) |
| Creatine | mg/dL | 0.8 (0.1) | 0.9 (0.1) | 0.8 (0.2) | 0.7 (0.1) | 0.8 (0.1) | 0.8 (0.1) | 0.8 (0.2) | 0.8 (0.2) | 0.7 (0.1) |
| Uric Acid | mg/dL | 0.2 (0) | 0.2 (0) | 0.2 (0) | 0.2 (0) | 0.2 (0) | 0.2 (0) | 0.2 (0) | 0.2 (0) | 0.2 (0) |
| Albumin | g/dL | 4 (0.1) | 3.7 (0.2) | 3.8 (0.2) | 4.1 (0.3) | 3.7 (0.2) | 3.8 (0.2) | 4.1 (0.1) | 4 (0.1) | 4 (0.2) |
| Total Protein | g/dL | 7.1 (0.3) | 6.5 (0.3) | 6.7 (0.2) | 7.2 (0.2) | 6.4 (0.1) | 6.7 (0.2) | 7.1 (0.2) | 6.9 (0.1) | 7 (0.3) |
| Cholesterol | mg/dL | 100.3 (22.8) | 84.3 (20.6) | 96 (12.2) | 110.3 (40.9) | 85.7 (27.6) | 90.3 (12.7) | 86.3 (12.9) | 79.7 (14.5) | 85 (10.5) |
| Triglycerides | mg/dL | 124.7 (116) | 118.7 (119.4) | 67 (60.2) | 69.3 (46.7) | 84.7 (70.1) | 71.8 (37.4) | 80.3 (47.4) | 112 (103.9) | 79.7 (51.7) |
| Alkaline Phosphatase | U/L | 115 (44.2) | 108.3 (43.6) | 100.7 (42) | 102 (36) | 95.7 (29.5) | 84.5 (30.1) | 104 (47.8) | 107.3 (44.7) | 100.7 (37.9) |
| ALT/GPT | U/L | 51.3 (17.9) | 46.3 (18.1) | 54.3 (13.8) | 35 (3.5) | 38 (4.6) | 45 (8.8) | 38.7 (7.5) | 42.7 (4.9) | 40.7 (15.8) |
| AST/GOT | U/L | 44 (6.6) | 57.3 (20.8) | 33.7 (6.4) | 39.7 (5) | 69.7 (11.2) | 52.8 (20.3) | 35 (4) | 47.3 (6) | 27 (3.5) |
| Amylase | U/L | 362 (96.6) | 348.7 (86) | 353.7 (95.7) | 370 (125.7) | 369.3 (88.3) | 346.3 (105.4) | 381.3 (139.1) | 453.3 (227.9) | 354 (112.3) |
| Total CK | U/L | 1314 (1207) | 1750.7 (1094.1) | 496 (117.1) | 366.3 (157.7) | 2418.3 (2391) | 2027.3 (1545.7) | 669.7 (19.2) | 1578.3 (299.5) | 598.7 (308.1) |
| LD | U/L | 941 (273.8) | 1005.7 (707.3) | 606.7 (370.7) | 915.7 (148.5) | 1223.3 (218.7) | 901.5 (384) | 616 (118.5) | 603 (146.5) | 376.3 (119.6) |
| Total Bilirubin | mg/dL | 0.2 (0) | 0.2 (0) | 0.2 (0) | 0.2 (0) | 0.2 (0) | 0.2 (0) | 0.2 (0) | 0.2 (0) | 0.2 (0) |
| Direct Bilirubin | mg/dL | 0.2 (0) | 0.2 (0) | 0.2 (0) | 0.2 (0) | 0.2 (0) | 0.2 (0) | 0.2 (0) | 0.2 (0) | 0.2 (0) |
| Tempurature | C | 99.2(0.6) | 101(0.2) |  | 100.9(0.6) | 101.2(1.2) |  | 100.6(0.5) | 100.1(0.9) |  |
