# Supplementary material for: CpG ODN D35 improves the response to abbreviated low-dose pentavalent antimonial treatment in non-human primate model of cutaneous leishmaniasis
Source: PLoS Negl Trop Dis. 2020 Feb 28;14(2):e0008050. doi: 10.1371/journal.pntd.0008050 (PMC7075640; doi:10.1371/journal.pntd.0008050)
Supplement: S4 Table — (DOCX) [file pntd.0008050.s013.docx]

| Supplementary Table III Genes 2-fold increased over baseline saline. | | | | | |
| --- | --- | --- | --- | --- | --- |
| Gene ID | probe.ID | Log2 fold change (mean) | std error | P-value | Corrected P value |
| ADA | XM_001109908.2:1305 | 1.22 | 0.286 | 0.0009 | 0.0127 |
| APOE | XM_005589553.1:498 | -1.19 | 0.187 | 0.0000 | 0.000501 |
| APP | XM_002803170.1:1684 | -1.21 | 0.195 | 0.0000 | 0.000633 |
| ARG2 | NM_001261253.1:529 | 1.22 | 0.337 | 0.0032 | 0.0379 |
| ARHGDIB | XM_005570240.1:356 | 1.8 | 0.22 | 0.0000 | 6.08E-05 |
| B2M | NM_001047137.1:25 | 2.41 | 0.171 | 0.0000 | 4.89E-07 |
| BAD | XM_005577426.2:425 | -3.12 | 0.841 | 0.0026 | 0.0317 |
| BATF | XM_005561820.1:826 | 3.38 | 0.413 | 0.0000 | 6.04E-05 |
| BATF3 | XM_005540791.1:296 | 3.65 | 0.383 | 0.0000 | 1.47E-05 |
| BCL3 | XM_001109319.2:329 | 2.09 | 0.246 | 0.0000 | 4.26E-05 |
| BID | XM_002798280.1:1164 | 1.68 | 0.245 | 0.0000 | 0.000279 |
| BLNK | XM_001100700.2:975 | 1.17 | 0.15 | 0.0000 | 9.14E-05 |
| BST2 | NM_001161666.1:64 | 3.6 | 0.179 | 0.0000 | 3.20E-08 |
| BTK | XM_002806330.1:1175 | 1.5 | 0.268 | 0.0001 | 0.00154 |
| BTLA | XM_005548166.1:1134 | 2.04 | 0.363 | 0.0001 | 0.0015 |
| C1QA | XM_001101933.2:476 | 3.32 | 0.207 | 0.0000 | 1.91E-07 |
| C1QB | XM_005544500.1:562 | 2.77 | 0.139 | 0.0000 | 3.20E-08 |
| C2 | XM_005553451.1:1121 | 1.81 | 0.163 | 0.0000 | 4.24E-06 |
| C3 | XM_001091921.2:3510 | 3.72 | 0.539 | 0.0000 | 0.000264 |
| C3AR1 | XM_001113373.1:564 | 3.11 | 0.221 | 0.0000 | 4.89E-07 |
| C4A | XM_002805966.1:1627 | 2.09 | 0.252 | 0.0000 | 5.45E-05 |
| C4B | XM_005553435.1:1550 | 2.26 | 0.226 | 0.0000 | 1.01E-05 |
| C5aR1 | XM_005589697.2:1207 | 2.13 | 0.194 | 0.0000 | 4.73E-06 |
| CARD9 | XM_001095549.2:1126 | 1.77 | 0.363 | 0.0003 | 0.00468 |
| CASP1 | XM_001094943.2:1600 | 1.96 | 0.236 | 0.0000 | 5.09E-05 |
| CASP10 | XM_001097804.2:1184 | 1.59 | 0.178 | 0.0000 | 2.78E-05 |
| CASP8 | XM_001091080.2:375 | 1.53 | 0.151 | 0.0000 | 9.93E-06 |
| CCL11 | NM_001032874.1:160 | 5.49 | 0.511 | 0.0000 | 5.57E-06 |
| CCL13 | XM_001113462.2:177 | -1.43 | 0.341 | 0.0011 | 0.0143 |
| CCL15 | XM_002800380.1:156 | 2.66 | 0.488 | 0.0001 | 0.00197 |
| CCL19 | NM_001032959.1:58 | 3.21 | 0.493 | 0.0000 | 0.000422 |
| CCL2 | NM_001032821.1:187 | 5.94 | 0.488 | 0.0000 | 1.91E-06 |
| CCL20 | NM_001032854.1:28 | 1.8 | 0.462 | 0.0019 | 0.0234 |
| CCL3 | NM_001034200.1:124 | 5.51 | 0.468 | 0.0000 | 2.54E-06 |
| CCL5 | NM_001032850.1:170 | 4.55 | 0.453 | 0.0000 | 1.01E-05 |
| CCL7 | XM_001113381.2:78 | 3.87 | 0.476 | 0.0000 | 6.31E-05 |
| CCL8 | NM_001032851.1:42 | 5.24 | 0.646 | 0.0000 | 6.39E-05 |
| CCR1 | NM_001032858.1:466 | 3.84 | 0.284 | 0.0000 | 7.36E-07 |
| CCR2 | NM_001032806.1:40 | 1.9 | 0.291 | 0.0000 | 0.00042 |
| CCR3 | NM_001047140.1:543 | 3.23 | 0.662 | 0.0003 | 0.00461 |
| CCR5 | XM_005546902.1:1188 | 4.53 | 0.436 | 0.0000 | 7.57E-06 |
| CCR7 | NM_001032884.1:178 | 3.55 | 0.541 | 0.0000 | 0.000406 |
| CCRL1 | XM_005545781.1:1286 | 1.93 | 0.389 | 0.0003 | 0.00406 |
| CCRL2 | XM_001103165.2:488 | 2.94 | 0.473 | 0.0000 | 0.000636 |
| CD14 | NM_001130433.1:517 | 1.88 | 0.254 | 0.0000 | 0.000141 |
| CD160 | XM_005542122.1:545 | 2.42 | 0.525 | 0.0005 | 0.00716 |
| CD163 | XM_001118361.2:1059 | 3.03 | 0.233 | 0.0000 | 1.03E-06 |
| CD19 | XM_001103043.2:655 | 2.06 | 0.494 | 0.0011 | 0.0149 |
| CD1A | XM_005595551.1:2397 | -1.84 | 0.489 | 0.0024 | 0.0293 |
| CD2 | NM_001283777.1:636 | 2.69 | 0.541 | 0.0003 | 0.00406 |
| CD247 | NM_001077423.1:191 | 3.07 | 0.545 | 0.0001 | 0.0015 |
| CD27 | XM_001104337.2:745 | 2.63 | 0.406 | 0.0000 | 0.00044 |
| CD274 | NM_001083889.1:505 | 6.04 | 0.526 | 0.0000 | 3.14E-06 |
| CD28 | NM_001042641.2:145 | 3.27 | 0.461 | 0.0000 | 0.000216 |
| CD34 | XM_005540699.1:669 | -1.52 | 0.25 | 0.0000 | 0.000789 |
| CD36 | NM_001032913.1:1240 | -2.04 | 0.404 | 0.0002 | 0.00349 |
| CD38 | XM_005554514.1:1197 | 5.96 | 0.38 | 0.0000 | 2.35E-07 |
| CD3D | NM_001287688.1:129 | 3.61 | 0.512 | 0.0000 | 0.000221 |
| CD3E | NM_001283615.1:236 | 2.76 | 0.396 | 0.0000 | 0.000251 |
| CD3G | NM_001283910.1:263 | 2.51 | 0.395 | 0.0000 | 0.000526 |
| CD4 | NM_001042662.1:796 | 1.61 | 0.127 | 0.0000 | 1.42E-06 |
| CD40 | XM_001104255.1:180 | 2.2 | 0.327 | 0.0000 | 0.000332 |
| CD40LG | NM_001032839.1:669 | 2.09 | 0.381 | 0.0001 | 0.00184 |
| CD48 | XM_005541239.1:597 | 2.87 | 0.299 | 0.0000 | 1.41E-05 |
| CD5 | XM_005578865.1:1771 | 1.84 | 0.413 | 0.0007 | 0.00934 |
| CD53 | XM_005542401.1:734 | 3.34 | 0.283 | 0.0000 | 2.51E-06 |
| CD6 | XM_002799538.1:945 | 1.83 | 0.409 | 0.0006 | 0.00894 |
| CD68 | XM_005582776.1:980 | 2.28 | 0.151 | 0.0000 | 3.09E-07 |
| CD69 | XM_005570089.2:434 | 3.23 | 0.575 | 0.0001 | 0.00151 |
| CD7 | XM_001113810.2:180 | 4.65 | 0.477 | 0.0000 | 1.29E-05 |
| CD74 | XM_001099491.2:702 | 2.8 | 0.24 | 0.0000 | 2.69E-06 |
| CD79A | XM_005589419.1:281 | 2.74 | 0.356 | 0.0000 | 0.000102 |
| CD79B | XM_005584687.1:260 | 1.41 | 0.344 | 0.0012 | 0.0163 |
| CD80 | NM_001042642.1:320 | 4.83 | 0.491 | 0.0000 | 1.20E-05 |
| CD83 | XM_005553991.1:694 | 2.77 | 0.338 | 0.0000 | 5.99E-05 |
| CD86 | NM_001042644.1:210 | 3.04 | 0.268 | 0.0000 | 3.50E-06 |
| CD8A | XM_001092778.2:630 | 3.02 | 0.484 | 0.0000 | 0.000622 |
| CD8B | XM_005575360.1:388 | 1.51 | 0.339 | 0.0007 | 0.00929 |
| CD9 | XM_005569889.1:719 | -1.29 | 0.249 | 0.0002 | 0.00286 |
| CD97 | XM_002801104.1:2191 | 1.5 | 0.139 | 0.0000 | 5.37E-06 |
| CD99 | XM_005592879.1:540 | -1.09 | 0.124 | 0.0000 | 3.02E-05 |
| CDKN1A | XM_005553160.1:259 | 1.05 | 0.201 | 0.0002 | 0.00268 |
| CEACAM3 | XM_015442082.1:1254 | 3.59 | 0.457 | 0.0000 | 8.70E-05 |
| CFB | XM_001113553.2:3014 | 4.45 | 0.416 | 0.0000 | 5.88E-06 |
| CFD | XM_001117186.2:846 | -2.14 | 0.311 | 0.0000 | 0.00027 |
| CFP | XM_005593429.1:1073 | 1.32 | 0.208 | 0.0000 | 0.000549 |
| CIITA | XM_001103518.2:558 | 3.74 | 0.312 | 0.0000 | 2.24E-06 |
| CISH | XM_001097616.2:438 | 1.7 | 0.294 | 0.0001 | 0.00121 |
| CLEC4A | XM_001113405.2:190 | 3.37 | 0.356 | 0.0000 | 1.61E-05 |
| CLEC5A | XM_001085243.2:595 | 1.9 | 0.492 | 0.0019 | 0.0244 |
| CLEC6A | XM_005570039.1:339 | 3.71 | 0.599 | 0.0000 | 0.000657 |
| CLEC7A | NM_001032943.1:89 | 2.65 | 0.395 | 0.0000 | 0.000336 |
| CMA1 | NM_001285192.1:191 | 1.01 | 0.189 | 0.0001 | 0.00228 |
| CMKLR1 | XM_005572139.1:3939 | 1.77 | 0.17 | 0.0000 | 7.36E-06 |
| CR1 | XM_002801945.1:2925 | 1.63 | 0.381 | 0.0009 | 0.0121 |
| CR2 | XM_002801946.1:2862 | 1.86 | 0.497 | 0.0025 | 0.0306 |
| CSF1R | XM_001107711.2:2036 | 1.73 | 0.194 | 0.0000 | 2.76E-05 |
| CSF2RB | XM_001086084.2:1149 | 3.56 | 0.478 | 0.0000 | 0.000136 |
| CSF3R | XM_005543945.1:2291 | 4.42 | 0.67 | 0.0000 | 0.000382 |
| CTLA4 | NM_001044739.1:364 | 2.73 | 0.403 | 0.0000 | 0.000305 |
| CTSC | NM_001195651.1:1020 | 2.21 | 0.276 | 0.0000 | 7.34E-05 |
| CTSS | XM_005541969.1:751 | 2.46 | 0.176 | 0.0000 | 5.26E-07 |
| CXCL10 | NM_001032892.1:169 | 9.99 | 0.645 | 0.0000 | 2.46E-07 |
| CXCL11 | XM_005555011.1:254 | 8.77 | 0.422 | 0.0000 | 3.20E-08 |
| CXCL2 | XM_001092240.2:156 | 3.43 | 0.339 | 0.0000 | 9.56E-06 |
| CXCL3 | NM_001032879.1:202 | 4.52 | 0.321 | 0.0000 | 4.89E-07 |
| CXCL6 | XM_005555086.1:565 | 2.43 | 0.583 | 0.0011 | 0.0148 |
| CXCL9 | NM_001032936.1:60 | 9.72 | 0.463 | 0.0000 | 3.20E-08 |
| CXCR3 | XM_005593931.1:680 | 3.14 | 0.442 | 0.0000 | 0.000214 |
| CXCR4 | NM_001042645.1:610 | 1.74 | 0.258 | 0.0000 | 0.000325 |
| CYBB | XM_001083654.2:1670 | 5.04 | 0.244 | 0.0000 | 3.20E-08 |
| CYSLTR2 | XM_005585841.1:592 | 1.18 | 0.309 | 0.0021 | 0.0263 |
| DDX58 | XM_005581558.1:1793 | 2.91 | 0.34 | 0.0000 | 4.06E-05 |
| DEFB1 | XM_005562538.1:180 | -1.1 | 0.292 | 0.0024 | 0.0293 |
| DEFB4A | XM_005562564.1:201 | 4.22 | 0.776 | 0.0001 | 0.00197 |
| DOCK9 | XM_005586152.1:1971 | -1.13 | 0.223 | 0.0002 | 0.00334 |
| EBI3 | XM_001118027.2:119 | 1.86 | 0.386 | 0.0003 | 0.0052 |
| EIF2AK2 | XM_015433780.1:1390 | 1.81 | 0.251 | 0.0000 | 0.000185 |
| EOMES | XM_005545584.1:1990 | 3.55 | 0.512 | 0.0000 | 0.000253 |
| ETS1 | XM_005580142.1:2883 | 1.35 | 0.158 | 0.0000 | 4.07E-05 |
| FAS | NM_001032933.1:797 | 1.62 | 0.215 | 0.0000 | 0.000124 |
| FASLG | NM_001032838.1:495 | 2.5 | 0.388 | 0.0000 | 0.000472 |
| FCAR | NM_001039950.1:390 | 3.4 | 0.374 | 0.0000 | 2.38E-05 |
| FCER1A | XM_005541313.1:452 | -1.37 | 0.33 | 0.0012 | 0.0154 |
| FCER1G | XM_005541196.1:395 | 2.17 | 0.167 | 0.0000 | 1.03E-06 |
| FCGR1A | NM_001284040.1:867 | 4.23 | 0.408 | 0.0000 | 7.57E-06 |
| FCGR2A | XM_001118066.2:766 | 2.51 | 0.333 | 0.0000 | 0.000124 |
| FCGR3 | NM_001283192.1:339 | 5.63 | 0.48 | 0.0000 | 2.58E-06 |
| FEZ1 | NM_001261621.2:516 | -1.58 | 0.358 | 0.0007 | 0.01 |
| GATA3 | XM_005564600.1:1760 | -1.58 | 0.275 | 0.0001 | 0.00129 |
| GBP1 | XM_005542801.1:1863 | 6.99 | 0.367 | 0.0000 | 5.09E-08 |
| GFI1 | XM_005542722.1:1019 | 1.39 | 0.385 | 0.0032 | 0.0378 |
| GNLY | XM_005575397.1:801 | 4.72 | 0.488 | 0.0000 | 1.35E-05 |
| GPR183 | XM_005586155.1:427 | 3.15 | 0.289 | 0.0000 | 5.01E-06 |
| GRB2 | XM_001095326.2:585 | 1.34 | 0.152 | 0.0000 | 2.99E-05 |
| GTF3C1 | XM_005591522.1:4642 | -1.02 | 0.14 | 0.0000 | 0.000168 |
| GZMA | XM_001097639.2:645 | 5.77 | 0.64 | 0.0000 | 2.55E-05 |
| GZMB | XM_005560998.1:576 | 7.54 | 0.522 | 0.0000 | 4.43E-07 |
| GZMH | XM_005560994.1:163 | 4.65 | 0.782 | 0.0000 | 0.000947 |
| GZMK | XM_005556895.1:484 | 4.58 | 0.468 | 0.0000 | 1.24E-05 |
| HAVCR2 | XM_005558381.1:529 | 3.79 | 0.263 | 0.0000 | 4.49E-07 |
| HIF1A | XM_005561440.1:2618 | 1.42 | 0.151 | 0.0000 | 1.63E-05 |
| HLA-A | NM_001171838.1:235 | 2.22 | 0.185 | 0.0000 | 2.18E-06 |
| HLA-B | NM_001114964.1:228 | 2.15 | 0.132 | 0.0000 | 1.86E-07 |
| HLA-C | NM_001048245.1:230 | 1.6 | 0.342 | 0.0004 | 0.00637 |
| HLA-DMA | XM_005553355.1:281 | 2.42 | 0.197 | 0.0000 | 1.79E-06 |
| HLA-DMB | XM_002803688.1:1486 | 2.18 | 0.247 | 0.0000 | 3.03E-05 |
| HLA-DOB | XM_005553370.1:723 | 2.32 | 0.311 | 0.0000 | 0.000136 |
| HLA-DPA1 | NM_001048247.1:214 | 3.09 | 0.209 | 0.0000 | 3.55E-07 |
| HLA-DPB1 | XM_005553343.1:545 | 2.65 | 0.275 | 0.0000 | 1.39E-05 |
| HLA-DQA1 | NM_001285343.1:429 | 3.01 | 0.298 | 0.0000 | 9.56E-06 |
| HLA-DQB1 | NM_001283172.1:428 | 2.76 | 0.27 | 0.0000 | 8.92E-06 |
| HLA-DRA | NM_001134298.1:360 | 3.02 | 0.255 | 0.0000 | 2.51E-06 |
| HLA-DRB1 | NM_001284641.1:634 | 2.76 | 0.233 | 0.0000 | 2.50E-06 |
| HSH2D | XM_005588318.1:1049 | 2.25 | 0.45 | 0.0002 | 0.00393 |
| HSPB1 | NM_001260949.2:416 | -1.15 | 0.28 | 0.0012 | 0.0159 |
| ICAM1 | NM_001047135.1:1170 | 3.55 | 0.251 | 0.0000 | 4.89E-07 |
| ICAM2 | NM_001195389.1:825 | 1.16 | 0.174 | 0.0000 | 0.000346 |
| ICAM3 | NM_001195749.1:233 | 2.06 | 0.314 | 0.0000 | 0.000407 |
| ICOS | XM_005574018.1:214 | 3.31 | 0.359 | 0.0000 | 2.05E-05 |
| ICOSLG | XM_002803112.1:150 | -1.11 | 0.191 | 0.0001 | 0.00117 |
| IDO1 | NM_001077483.1:133 | 10.1 | 0.495 | 0.0000 | 3.20E-08 |
| IDO2 | XM_001095833.2:363 | 2.25 | 0.517 | 0.0008 | 0.0109 |
| IFI16 | XM_005541319.1:858 | 1.07 | 0.18 | 0.0000 | 0.000964 |
| IFI35 | XM_001112861.2:789 | 2.24 | 0.275 | 0.0000 | 6.30E-05 |
| IFI44 | XM_005542938.1:626 | 3.22 | 0.309 | 0.0000 | 7.36E-06 |
| IFIH1 | NM_001047123.1:655 | 2.86 | 0.296 | 0.0000 | 1.36E-05 |
| IFIT1 | NM_001287726.1:1154 | 3.44 | 0.415 | 0.0000 | 5.19E-05 |
| IFIT2 | XM_001086302.2:1204 | 3.96 | 0.414 | 0.0000 | 1.45E-05 |
| IFIT3 | XM_005565907.1:776 | 3.75 | 0.387 | 0.0000 | 1.35E-05 |
| IFITM1 | XM_005576718.1:454 | 2.46 | 0.214 | 0.0000 | 3.14E-06 |
| IFNAR2 | XM_001092342.2:1272 | 2.37 | 0.261 | 0.0000 | 2.38E-05 |
| IFNG | NM_001287657.1:358 | 5.07 | 0.746 | 0.0000 | 0.000303 |
| IFNGR2 | XM_005548806.2:830 | 2.05 | 0.18 | 0.0000 | 3.48E-06 |
| IKBKE | XM_005540634.1:1473 | 1.48 | 0.33 | 0.0006 | 0.0088 |
| IKZF1 | XM_005549595.1:528 | 2.89 | 0.282 | 0.0000 | 8.79E-06 |
| IKZF3 | XM_005584045.1:1828 | 3.34 | 0.499 | 0.0000 | 0.00034 |
| IL10 | XM_005540653.1:448 | 2.31 | 0.309 | 0.0000 | 0.000132 |
| IL10RA | XM_001092736.2:525 | 2.41 | 0.297 | 0.0000 | 6.26E-05 |
| IL10RB | XM_005548811.1:270 | 1.37 | 0.184 | 0.0000 | 0.00014 |
| IL11RA | XM_005581441.1:642 | -1.84 | 0.375 | 0.0003 | 0.00448 |
| IL12RB1 | XM_001115186.2:1312 | 2.92 | 0.616 | 0.0004 | 0.00581 |
| IL12RB2 | XM_001094235.1:882 | 1.78 | 0.261 | 0.0000 | 0.000293 |
| IL13RA1 | XM_002806369.1:670 | 1.04 | 0.151 | 0.0000 | 0.000265 |
| IL15 | NM_001044731.1:113 | 1.76 | 0.208 | 0.0000 | 4.34E-05 |
| IL18R1 | XM_002799354.1:1245 | 2.19 | 0.372 | 0.0001 | 0.00101 |
| IL18RAP | XM_005575166.1:1147 | 3.77 | 0.591 | 0.0000 | 0.000505 |
| IL1B | NM_001042756.1:391 | 4.74 | 0.517 | 0.0000 | 2.18E-05 |
| IL1RN | XM_005575305.1:499 | 2.54 | 0.413 | 0.0000 | 0.000688 |
| IL21 | XM_005555864.1:148 | 2.97 | 0.303 | 0.0000 | 1.24E-05 |
| IL21R | XM_001094102.2:295 | 4.28 | 0.396 | 0.0000 | 5.37E-06 |
| IL22RA2 | XM_001099782.2:822 | 2.11 | 0.567 | 0.0026 | 0.0317 |
| IL27 | XM_001097165.2:172 | 2.56 | 0.379 | 0.0000 | 0.000316 |
| IL2RA | NM_001032917.1:652 | 4.62 | 0.61 | 0.0000 | 0.000121 |
| IL2RB | XM_001085792.2:202 | 4.14 | 0.39 | 0.0000 | 6.22E-06 |
| IL2RG | NM_001035529.1:645 | 2.99 | 0.385 | 0.0000 | 9.45E-05 |
| IL4R | XM_001093763.2:655 | 1.75 | 0.219 | 0.0000 | 7.49E-05 |
| IL6 | NM_001042733.1:139 | 6.76 | 0.434 | 0.0000 | 2.42E-07 |
| IL7R | NM_001284908.1:522 | 3.05 | 0.439 | 0.0000 | 0.000253 |
| IL8 | NM_001032965.1:125 | 3.75 | 0.978 | 0.0021 | 0.026 |
| IRAK2 | XM_001090790.2:595 | 1.05 | 0.269 | 0.0018 | 0.0225 |
| IRF1 | XM_001104048.1:179 | 4.5 | 0.297 | 0.0000 | 2.98E-07 |
| IRF7 | NM_001136100.1:1398 | 5.05 | 0.372 | 0.0000 | 7.15E-07 |
| IRF8 | XM_005592710.1:1266 | 4.6 | 0.308 | 0.0000 | 3.22E-07 |
| ISG20 | XM_005560453.2:437 | 3.79 | 0.427 | 0.0000 | 2.85E-05 |
| ITGA4 | XM_001100929.2:2035 | 2.43 | 0.285 | 0.0000 | 4.11E-05 |
| ITGAL | XM_001100800.2:1305 | 3.93 | 0.478 | 0.0000 | 5.72E-05 |
| ITGAM | XM_005591740.1:1671 | 2.84 | 0.321 | 0.0000 | 2.92E-05 |
| ITGAX | XM_005591741.1:2025 | 2.86 | 0.338 | 0.0000 | 4.40E-05 |
| ITGB2 | XM_005548517.1:853 | 3.42 | 0.279 | 0.0000 | 1.83E-06 |
| JAK2 | XM_005581792.1:3097 | 1.75 | 0.21 | 0.0000 | 5.07E-05 |
| JAK3 | XM_001115037.2:2516 | 3.46 | 0.479 | 0.0000 | 0.000179 |
| KIR3DL1 | NM_001193463.1:758 | 2.12 | 0.51 | 0.0011 | 0.0151 |
| KLRC1 | NM_001284177.1:95 | 3.9 | 0.554 | 0.0000 | 0.000222 |
| KLRC3 | XM_005595216.1:692 | 3.91 | 0.598 | 0.0000 | 0.000407 |
| KLRD1 | NM_001032828.1:365 | 4.54 | 0.48 | 0.0000 | 1.61E-05 |
| KLRF1 | NM_001285324.1:514 | 3.13 | 0.728 | 0.0009 | 0.012 |
| KLRF2 | XM_005570092.1:616 | 2.47 | 0.555 | 0.0007 | 0.00929 |
| KLRK1 | NM_001283284.1:374 | 3.91 | 0.374 | 0.0000 | 7.28E-06 |
| LAG3 | XM_005569954.1:1283 | 2.59 | 0.469 | 0.0001 | 0.00174 |
| LAIR1 | XM_005590317.1:341 | 3.56 | 0.336 | 0.0000 | 6.22E-06 |
| LAIR2 | XM_005590334.1:128 | 2.22 | 0.433 | 0.0002 | 0.00311 |
| LAMP3 | XM_005546488.1:987 | 2.6 | 0.579 | 0.0006 | 0.00883 |
| LCK | XM_002802327.1:263 | 2.71 | 0.438 | 0.0000 | 0.000659 |
| LCP2 | XM_005558492.1:895 | 3.44 | 0.395 | 0.0000 | 3.40E-05 |
| LIF | XM_005567631.1:2704 | 2.31 | 0.418 | 0.0001 | 0.00175 |
| LILRA2 | XM_005596068.1:724 | 3.03 | 0.523 | 0.0001 | 0.00119 |
| LILRA3 | NM_001040675.1:230 | 3.19 | 0.329 | 0.0000 | 1.34E-05 |
| LILRA4 | XM_005590289.1:685 | 4.46 | 0.576 | 0.0000 | 9.80E-05 |
| LILRB1 | NM_001040672.1:52 | 2.73 | 0.407 | 0.0000 | 0.000334 |
| LILRB3 | XM_005590303.1:1479 | 3.11 | 0.443 | 0.0000 | 0.000233 |
| LILRB4 | NM_001040676.1:635 | 3.93 | 0.629 | 0.0000 | 0.000622 |
| LIMK1 | XM_005595495.1:2173 | 1.44 | 0.311 | 0.0005 | 0.00709 |
| LITAF | XM_005591267.1:620 | 1.4 | 0.178 | 0.0000 | 9.04E-05 |
| LTB | NM_001047150.1:222 | 2.01 | 0.572 | 0.0038 | 0.0448 |
| LTF | XM_005546908.1:1534 | 3.14 | 0.605 | 0.0002 | 0.00289 |
| LY96 | NM_001130432.1:327 | 1.9 | 0.259 | 0.0000 | 0.000154 |
| MAP4K1 | XM_001082963.2:300 | 2.56 | 0.266 | 0.0000 | 1.37E-05 |
| MME | XM_001105564.1:1261 | -1.8 | 0.433 | 0.0011 | 0.0149 |
| MMP3 | XM_001098400.2:935 | 3.9 | 0.681 | 0.0001 | 0.0013 |
| MMP9 | XM_001104871.2:1515 | 4.68 | 0.728 | 0.0000 | 0.000477 |
| MR1 | NM_001284968.1:765 | 1.55 | 0.185 | 0.0000 | 4.58E-05 |
| MRC1 | XM_005564743.1:1361 | 1.19 | 0.198 | 0.0000 | 0.000845 |
| MS4A1 | XM_005577709.1:708 | 2.68 | 0.512 | 0.0002 | 0.00273 |
| MSR1 | XM_001097884.2:1125 | 2.19 | 0.345 | 0.0000 | 0.000528 |
| MX1 | NM_001079693.1:1034 | 4.12 | 0.479 | 0.0000 | 3.86E-05 |
| MX2 | XM_005548647.1:1483 | 4.32 | 0.381 | 0.0000 | 3.50E-06 |
| MYD88 | NM_001130681.1:602 | 2.13 | 0.214 | 0.0000 | 1.04E-05 |
| NCF4 | XM_005567397.1:536 | 2.78 | 0.422 | 0.0000 | 0.000383 |
| NFKB1 | XM_005555555.1:1074 | 1.06 | 0.131 | 0.0000 | 6.86E-05 |
| NFKB2 | XM_001104566.2:905 | 1.56 | 0.233 | 0.0000 | 0.000338 |
| NLRP3 | XM_005539576.1:2196 | 2.11 | 0.372 | 0.0001 | 0.00138 |
| NOD1 | XM_005549884.1:2719 | 1.21 | 0.138 | 0.0000 | 3.40E-05 |
| NOD2 | XM_005591903.1:1212 | 1.69 | 0.291 | 0.0001 | 0.00112 |
| OAS1 | NM_001083949.1:731 | 3.67 | 0.425 | 0.0000 | 3.70E-05 |
| OAS2 | XM_005572308.1:515 | 4.79 | 0.408 | 0.0000 | 2.58E-06 |
| OAS3 | XM_005572307.1:2923 | 2.74 | 0.248 | 0.0000 | 4.33E-06 |
| OASL | XM_005572424.1:1339 | 4.64 | 0.521 | 0.0000 | 2.78E-05 |
| PDCD1 | NM_001114358.1:201 | 2.32 | 0.376 | 0.0000 | 0.000676 |
| PDCD1LG2 | NM_001083599.1:760 | 3.29 | 0.371 | 0.0000 | 2.88E-05 |
| PIGR | XM_001083307.2:2166 | 2.9 | 0.409 | 0.0000 | 0.000214 |
| PIK3C2G | XM_002807975.1:2245 | -1.75 | 0.43 | 0.0013 | 0.0171 |
| PLA2G2A | XM_005544567.1:484 | 5.04 | 0.716 | 0.0000 | 0.000223 |
| PLA2G6 | XM_002798388.1:2066 | -1.41 | 0.371 | 0.0022 | 0.0275 |
| PLAU | XM_005565444.1:1078 | 1.49 | 0.292 | 0.0002 | 0.00337 |
| PLAUR | XM_001106634.2:532 | 2.21 | 0.328 | 0.0000 | 0.000324 |
| PLCB1 | XM_001116017.1:715 | -1.58 | 0.288 | 0.0001 | 0.00187 |
| PML | NM_001042434.1:441 | 1.54 | 0.155 | 0.0000 | 1.04E-05 |
| POU2F2 | XM_002808195.1:1136 | 2.89 | 0.364 | 0.0000 | 8.02E-05 |
| PPARG | NM_001032860.1:980 | -1.32 | 0.298 | 0.0007 | 0.0099 |
| PPP1R12B | XM_001106168.2:540 | -1.18 | 0.157 | 0.0000 | 0.000122 |
| PRF1 | XM_005565623.1:1621 | 4.08 | 0.44 | 0.0000 | 1.96E-05 |
| PRKCB | XM_001095880.2:1530 | 2.04 | 0.345 | 0.0001 | 0.000997 |
| PSMB10 | XM_005592304.1:741 | 3.75 | 0.283 | 0.0000 | 8.83E-07 |
| PSMB8 | XM_005553359.1:807 | 3.65 | 0.214 | 0.0000 | 1.33E-07 |
| PSMB9 | NM_001283167.1:407 | 3.59 | 0.254 | 0.0000 | 4.89E-07 |
| PTAFR | XM_005544241.1:1072 | 1.27 | 0.239 | 0.0001 | 0.00238 |
| PTGER2 | XM_005561261.1:1979 | 1.85 | 0.413 | 0.0006 | 0.00904 |
| PTGIR | XM_005589661.1:920 | 2.31 | 0.306 | 0.0000 | 0.000122 |
| PTGS2 | XM_001107538.2:1343 | 4.14 | 0.549 | 0.0000 | 0.000124 |
| PTK2 | XM_001093060.2:843 | -1.14 | 0.182 | 0.0000 | 0.000622 |
| PTPN2 | XM_005587248.1:320 | 1.09 | 0.19 | 0.0001 | 0.00127 |
| PTPN22 | XM_005542274.1:2386 | 2.82 | 0.397 | 0.0000 | 0.000214 |
| PTPN6 | XM_005569995.1:662 | 1.82 | 0.236 | 0.0000 | 0.000104 |
| PTPRC | XM_005540333.1:1225 | 3.13 | 0.311 | 0.0000 | 1.01E-05 |
| RARRES3 | XM_001118373.2:324 | 6.95 | 0.389 | 0.0000 | 8.63E-08 |
| RELB | XM_001104559.2:724 | 1.97 | 0.295 | 0.0000 | 0.000356 |
| RIPK2 | XM_005563661.1:895 | 1.08 | 0.226 | 0.0004 | 0.00535 |
| RORC | XM_002801778.1:1471 | -1.48 | 0.316 | 0.0004 | 0.00653 |
| RUNX1 | XM_005548734.1:611 | 1.08 | 0.162 | 0.0000 | 0.000352 |
| S100A8 | XM_005541787.1:236 | 5.44 | 0.939 | 0.0001 | 0.00118 |
| S100A9 | XM_005541789.1:223 | 6.27 | 1.14 | 0.0001 | 0.00174 |
| SELE | XM_005539941.1:1616 | 4.2 | 0.42 | 0.0000 | 1.01E-05 |
| SELL | XM_005539939.1:626 | 4.26 | 0.442 | 0.0000 | 1.39E-05 |
| SELPLG | XM_005572152.1:1151 | 2.44 | 0.452 | 0.0001 | 0.0021 |
| SERPING1 | XM_001092271.2:1235 | 1.5 | 0.168 | 0.0000 | 2.85E-05 |
| SH2D1A | NM_001032837.1:135 | 3.21 | 0.425 | 0.0000 | 0.000123 |
| SIGLEC1 | XM_005568446.2:2406 | 2.63 | 0.208 | 0.0000 | 1.42E-06 |
| SLAMF1 | XM_001117605.2:526 | 2.74 | 0.395 | 0.0000 | 0.000253 |
| SLAMF6 | XM_005541247.1:658 | 3.78 | 0.672 | 0.0001 | 0.00151 |
| SLAMF7 | XM_001117618.2:1032 | 5.26 | 0.587 | 0.0000 | 2.70E-05 |
| SMAD5 | XM_005557849.1:212 | -1.39 | 0.196 | 0.0000 | 0.000216 |
| SOCS1 | XM_005591262.1:760 | 2.87 | 0.363 | 0.0000 | 8.18E-05 |
| SOCS3 | NM_001194326.1:599 | 4.35 | 0.353 | 0.0000 | 1.79E-06 |
| STAT1 | NM_001261614.1:1598 | 3.12 | 0.173 | 0.0000 | 8.61E-08 |
| STAT2 | XM_001115072.2:2435 | 1.91 | 0.189 | 0.0000 | 9.56E-06 |
| STAT4 | XM_001082561.2:2220 | 2.32 | 0.571 | 0.0013 | 0.0175 |
| STAT5A | XM_001109557.2:2414 | 1.39 | 0.19 | 0.0000 | 0.000159 |
| SYK | XM_005582224.1:1575 | 2.43 | 0.283 | 0.0000 | 3.87E-05 |
| TAGAP | XM_005551523.1:931 | 2.77 | 0.393 | 0.0000 | 0.000221 |
| TAP1 | XM_005553361.1:970 | 3.15 | 0.253 | 0.0000 | 1.64E-06 |
| TAP2 | XM_002803985.1:555 | 3 | 0.274 | 0.0000 | 4.88E-06 |
| TAPBP | XM_005553311.1:1042 | 1.8 | 0.119 | 0.0000 | 2.98E-07 |
| TARP | XM_001100492.2:245 | 2.49 | 0.507 | 0.0003 | 0.00448 |
| TBX21 | XM_005583510.1:744 | 5.04 | 0.446 | 0.0000 | 3.56E-06 |
| TCF7 | XM_005595987.1:820 | 1.83 | 0.436 | 0.0011 | 0.0143 |
| TFRC | XM_005545258.1:2141 | 1.03 | 0.176 | 0.0001 | 0.0011 |
| TGFB1 | XM_005589339.1:1170 | 1.37 | 0.183 | 0.0000 | 0.000132 |
| TGFB2 | XM_005540874.1:1913 | -1.54 | 0.297 | 0.0002 | 0.00289 |
| TGFB3 | XM_005561843.1:1701 | 1.07 | 0.204 | 0.0002 | 0.00269 |
| TICAM1 | NM_001287328.1:1134 | 1.07 | 0.204 | 0.0002 | 0.00262 |
| TIGIT | XM_005548101.1:411 | 2.77 | 0.569 | 0.0003 | 0.00477 |
| TLR1 | NM_001130424.1:487 | 2.92 | 0.309 | 0.0000 | 1.61E-05 |
| TLR2 | NM_001130425.1:40 | 4.34 | 0.265 | 0.0000 | 1.80E-07 |
| TLR4 | NM_001037092.1:2100 | 3.42 | 0.26 | 0.0000 | 9.43E-07 |
| TLR6 | NM_001130430.1:123 | 1.27 | 0.249 | 0.0002 | 0.00332 |
| TLR7 | NM_001130426.1:55 | 2.92 | 0.291 | 0.0000 | 1.01E-05 |
| TLR8 | NM_001130427.1:120 | 2.35 | 0.174 | 0.0000 | 7.36E-07 |
| TLR9 | NM_001130431.1:839 | 2.48 | 0.576 | 0.0008 | 0.0118 |
| TMEM173 | XM_005557935.1:904 | 2.34 | 0.145 | 0.0000 | 1.86E-07 |
| TNF | NM_001047149.1:195 | 3.1 | 0.503 | 0.0000 | 0.000686 |
| TNFAIP3 | NM_001283816.1:1043 | 2.73 | 0.353 | 0.0000 | 9.97E-05 |
| TNFAIP6 | XM_001083111.2:166 | 4.49 | 0.272 | 0.0000 | 1.77E-07 |
| TNFRSF13B | XM_005582995.1:465 | 1.77 | 0.508 | 0.0040 | 0.0479 |
| TNFRSF14 | NM_001043357.1:107 | 1.44 | 0.261 | 0.0001 | 0.00175 |
| TNFRSF18 | XM_005545123.2:503 | 2.2 | 0.541 | 0.0014 | 0.0175 |
| TNFRSF1B | XM_001105753.2:840 | 2.35 | 0.251 | 0.0000 | 1.76E-05 |
| TNFRSF4 | XM_005545122.1:233 | 2.13 | 0.424 | 0.0002 | 0.00378 |
| TNFRSF9 | XM_001096166.2:295 | 1.53 | 0.391 | 0.0018 | 0.0229 |
| TNFSF13 | XM_005582760.2:625 | 1.85 | 0.219 | 0.0000 | 4.40E-05 |
| TNFSF13B | XM_005586224.1:906 | 3.28 | 0.185 | 0.0000 | 8.63E-08 |
| TNFSF8 | XM_005580959.1:769 | 1.99 | 0.282 | 0.0000 | 0.000218 |
| TRAF1 | XM_001095862.2:983 | 2.89 | 0.384 | 0.0000 | 0.000124 |
| TRAF3 | XM_005562258.1:431 | 1.26 | 0.163 | 0.0000 | 0.000102 |
| TRAF4 | XM_001107048.2:1637 | 1.04 | 0.175 | 0.0000 | 0.000921 |
| TREM2 | XM_005553065.1:581 | 2.77 | 0.402 | 0.0000 | 0.000266 |
| TYROBP | XM_005588917.1:524 | 3.02 | 0.281 | 0.0000 | 5.50E-06 |
| VCAM1 | XM_005542580.1:955 | 1.64 | 0.306 | 0.0001 | 0.00225 |
| XCL1 | NM_001032947.1:18 | 5.06 | 0.526 | 0.0000 | 1.40E-05 |
| XCR1 | XM_001114260.1:745 | 1.8 | 0.491 | 0.0029 | 0.0346 |
| ZBTB16 | XM_005579695.1:1968 | -2.28 | 0.329 | 0.0000 | 0.000252 |
|  |  |  |  |  |  |
