# Supplementary material for: CpG ODN D35 improves the response to abbreviated low-dose pentavalent antimonial treatment in non-human primate model of cutaneous leishmaniasis
Source: PLoS Negl Trop Dis. 2020 Feb 28;14(2):e0008050. doi: 10.1371/journal.pntd.0008050 (PMC7075640; doi:10.1371/journal.pntd.0008050)
Supplement: S5 Table — (DOCX) [file pntd.0008050.s014.docx]

| Supplementary Table IV Genes 2-fold increase over baseline D35. | | | | | |
| --- | --- | --- | --- | --- | --- |
| Gene ID | probe.ID | Log2 fold change (mean) | std error | P-value | Corrected P value |
| ADA | NM_001043357.1:107 | 1.28 | 0.266 | 0.000335 | 0.00499 |
| APP | XM_001102910.2:1015 | -1.1 | 0.181 | 3.98E-05 | 0.000783 |
| ARG1 | XM_001090841.2:374 | -1.77 | 0.385 | 0.000513 | 0.00733 |
| ARHGDIB | XM_005590289.1:685 | 1.78 | 0.205 | 9.01E-07 | 3.35E-05 |
| B2M | XM_001113381.2:78 | 2.39 | 0.159 | 1.34E-09 | 3.21E-07 |
| BAD | XM_005595551.1:2397 | -3.66 | 0.781 | 0.000431 | 0.00628 |
| BATF | NM_001130681.1:602 | 3.25 | 0.384 | 1.21E-06 | 4.15E-05 |
| BATF3 | XM_005548647.1:1483 | 3.54 | 0.356 | 1.87E-07 | 1.03E-05 |
| BAX | XM_005554158.1:1545 | 1.09 | 0.148 | 5.44E-06 | 0.000147 |
| BCL3 | XM_001117186.2:846 | 2.03 | 0.229 | 7.08E-07 | 2.78E-05 |
| BID | XM_005553370.1:723 | 1.57 | 0.228 | 1.11E-05 | 0.000264 |
| BLNK | NM_001130430.1:123 | 1.3 | 0.14 | 3.97E-07 | 1.79E-05 |
| BST2 | XM_005583510.1:744 | 3.63 | 0.166 | 1.26E-11 | 1.59E-08 |
| BTK | XM_005593429.1:1073 | 1.64 | 0.249 | 1.80E-05 | 0.000408 |
| BTLA | XM_002798280.1:1164 | 1.98 | 0.337 | 5.41E-05 | 0.00102 |
| C1QA | NM_001283172.1:428 | 3.34 | 0.193 | 2.23E-10 | 9.29E-08 |
| C1QB | XM_005565907.1:776 | 2.94 | 0.129 | 7.22E-12 | 1.59E-08 |
| C2 | XM_005595216.1:692 | 1.97 | 0.151 | 7.84E-09 | 1.02E-06 |
| C3 | XM_001092736.2:525 | 3.07 | 0.501 | 3.66E-05 | 0.000738 |
| C3AR1 | NM_001130432.1:327 | 2.77 | 0.205 | 5.01E-09 | 7.68E-07 |
| C4A | XM_005584241.1:1000 | 1.69 | 0.235 | 6.74E-06 | 0.000177 |
| C4B | XM_005562258.1:431 | 1.94 | 0.21 | 4.45E-07 | 1.92E-05 |
| C5aR1 | XM_005587248.1:320 | 1.82 | 0.18 | 1.54E-07 | 8.95E-06 |
| C7 | XM_005589911.1:713 | -2.02 | 0.391 | 0.000181 | 0.003 |
| CARD9 | XM_001114248.2:1870 | 1.66 | 0.337 | 0.000277 | 0.00425 |
| CASP1 | XM_002805966.1:1627 | 2.17 | 0.219 | 1.98E-07 | 1.06E-05 |
| CASP10 | XM_001107538.2:1343 | 1.39 | 0.166 | 1.31E-06 | 4.45E-05 |
| CASP8 | XM_005541663.1:1684 | 1.63 | 0.141 | 3.26E-08 | 3.00E-06 |
| CCBP2 | XM_001114260.1:745 | -1.09 | 0.219 | 0.000251 | 0.00396 |
| CCL11 | NM_001114964.1:228 | 5.4 | 0.475 | 3.94E-08 | 3.37E-06 |
| CCL13 | XM_005550046.1:1361 | -1.91 | 0.317 | 4.40E-05 | 0.000851 |
| CCL15 | XM_001092342.2:1272 | 2.48 | 0.454 | 0.000109 | 0.00191 |
| CCL19 | XM_005582760.2:625 | 3.28 | 0.458 | 7.27E-06 | 0.000189 |
| CCL2 | NM_001130425.1:40 | 5.05 | 0.453 | 5.07E-08 | 4.01E-06 |
| CCL3 | XM_001083111.2:166 | 4.67 | 0.435 | 7.83E-08 | 5.45E-06 |
| CCL5 | XM_005575397.1:801 | 3.47 | 0.421 | 1.62E-06 | 5.25E-05 |
| CCL7 | NM_001083889.1:505 | 3.47 | 0.443 | 2.83E-06 | 8.42E-05 |
| CCL8 | XM_005553311.1:1042 | 5.38 | 0.6 | 6.33E-07 | 2.52E-05 |
| CCR1 | XM_005589697.2:1207 | 3.25 | 0.264 | 1.55E-08 | 1.70E-06 |
| CCR2 | XM_001097165.2:172 | 1.76 | 0.271 | 1.98E-05 | 0.000438 |
| CCR5 | NM_001032874.1:160 | 3.65 | 0.406 | 5.97E-07 | 2.42E-05 |
| CCR7 | NM_001083949.1:731 | 3.06 | 0.503 | 3.80E-05 | 0.000759 |
| CCRL1 | XM_001098960.2:707 | 1.69 | 0.362 | 0.000433 | 0.00628 |
| CCRL2 | NM_001042756.1:391 | 2.12 | 0.439 | 0.00033 | 0.00493 |
| CD14 | XM_001095880.2:1530 | 1.76 | 0.236 | 4.85E-06 | 0.000136 |
| CD160 | XM_001104559.2:724 | 1.93 | 0.488 | 0.00162 | 0.0205 |
| CD163 | NM_001032837.1:135 | 3.03 | 0.216 | 3.19E-09 | 5.29E-07 |
| CD19 | XM_002806369.1:670 | 2.09 | 0.46 | 0.000553 | 0.00783 |
| CD1A | XM_005542722.1:1019 | -1.59 | 0.455 | 0.00401 | 0.0481 |
| CD2 | XM_005540791.1:296 | 3.22 | 0.503 | 2.30E-05 | 0.000501 |
| CD247 | XM_001094102.2:295 | 3.51 | 0.507 | 1.03E-05 | 0.000252 |
| CD27 | XM_005582224.1:1575 | 3.24 | 0.377 | 9.97E-07 | 3.61E-05 |
| CD274 | NM_001032892.1:169 | 5.36 | 0.489 | 6.09E-08 | 4.53E-06 |
| CD28 | XM_005591522.1:4642 | 2.86 | 0.429 | 1.51E-05 | 0.000347 |
| CD34 | XM_005541247.1:658 | -1.84 | 0.232 | 2.43E-06 | 7.44E-05 |
| CD36 | XM_005547856.1:2271 | -1.82 | 0.375 | 0.000315 | 0.00474 |
| CD38 | XM_001104048.1:179 | 5.3 | 0.353 | 1.37E-09 | 3.21E-07 |
| CD3D | NM_001032947.1:18 | 3.39 | 0.476 | 7.84E-06 | 0.000199 |
| CD3E | XM_005555864.1:148 | 2.75 | 0.368 | 4.64E-06 | 0.00013 |
| CD3G | XM_005556895.1:484 | 2.62 | 0.367 | 7.67E-06 | 0.000197 |
| CD4 | XM_001091921.2:3510 | 1.66 | 0.118 | 3.08E-09 | 5.29E-07 |
| CD40 | XM_005553991.1:694 | 2.24 | 0.304 | 5.56E-06 | 0.00015 |
| CD40LG | NM_001283777.1:636 | 1.77 | 0.354 | 0.000247 | 0.00392 |
| CD48 | NM_001032851.1:42 | 2.84 | 0.278 | 1.40E-07 | 8.33E-06 |
| CD5 | XM_001095862.2:983 | 1.9 | 0.384 | 0.000271 | 0.00418 |
| CD53 | XM_001094943.2:1600 | 3.19 | 0.263 | 1.81E-08 | 1.87E-06 |
| CD6 | XM_005553014.1:1367 | 2.17 | 0.38 | 7.22E-05 | 0.00132 |
| CD68 | NM_001283910.1:263 | 2.13 | 0.141 | 1.23E-09 | 3.21E-07 |
| CD69 | XM_005586155.1:427 | 3.01 | 0.534 | 8.12E-05 | 0.00146 |
| CD7 | NM_001284641.1:634 | 3.86 | 0.444 | 8.90E-07 | 3.35E-05 |
| CD74 | XM_001095326.2:585 | 2.65 | 0.223 | 2.37E-08 | 2.26E-06 |
| CD79A | NM_001130433.1:517 | 2.28 | 0.331 | 1.09E-05 | 0.000259 |
| CD80 | XM_005572308.1:515 | 3.8 | 0.456 | 1.42E-06 | 4.70E-05 |
| CD83 | XM_005548734.1:611 | 2.33 | 0.315 | 5.09E-06 | 0.00014 |
| CD86 | NM_001287726.1:1154 | 2.44 | 0.249 | 2.28E-07 | 1.19E-05 |
| CD8A | XM_005544567.1:484 | 3.08 | 0.45 | 1.18E-05 | 0.000278 |
| CD8B | XM_005541319.1:858 | 1.99 | 0.315 | 2.69E-05 | 0.000577 |
| CD9 | NM_001032860.1:980 | -1.42 | 0.231 | 3.58E-05 | 0.000725 |
| CD97 | XM_001118066.2:766 | 1.24 | 0.129 | 2.82E-07 | 1.38E-05 |
| CD99 | XM_001118027.2:119 | -1.15 | 0.115 | 1.76E-07 | 9.77E-06 |
| CEACAM3 | NM_001034200.1:124 | 3.41 | 0.425 | 2.17E-06 | 6.82E-05 |
| CFB | XM_005592710.1:1266 | 4.94 | 0.386 | 9.79E-09 | 1.18E-06 |
| CFD | XM_005541985.1:2124 | -2.01 | 0.289 | 9.96E-06 | 0.000244 |
| CFP | XM_005591044.1:604 | 1.18 | 0.194 | 3.84E-05 | 0.000763 |
| CIITA | NM_001285343.1:429 | 3.11 | 0.29 | 7.82E-08 | 5.45E-06 |
| CISH | XM_005572152.1:1151 | 1.03 | 0.273 | 0.00239 | 0.0298 |
| CLEC4A | XM_005555555.1:1074 | 2.5 | 0.331 | 4.26E-06 | 0.000122 |
| CLEC6A | XM_005548806.2:830 | 3.66 | 0.557 | 1.77E-05 | 0.000403 |
| CLEC7A | XM_005574018.1:214 | 2.89 | 0.367 | 2.73E-06 | 8.15E-05 |
| CMKLR1 | XM_005581441.1:642 | 1.69 | 0.158 | 7.97E-08 | 5.45E-06 |
| CR1 | NM_001042645.1:610 | 1.6 | 0.354 | 0.000568 | 0.008 |
| CSF1R | XM_001113462.2:177 | 1.54 | 0.18 | 1.08E-06 | 3.86E-05 |
| CSF2RB | NM_001194326.1:599 | 3.44 | 0.444 | 3.20E-06 | 9.35E-05 |
| CSF3R | XM_001113373.1:564 | 4.22 | 0.622 | 1.29E-05 | 0.000301 |
| CTLA4 | XM_001100800.2:1305 | 2.42 | 0.374 | 2.09E-05 | 0.000459 |
| CTSC | XM_001113405.2:190 | 2.3 | 0.257 | 6.21E-07 | 2.49E-05 |
| CTSS | XM_002802327.1:263 | 2.17 | 0.163 | 6.03E-09 | 8.24E-07 |
| CXCL10 | XM_005544500.1:562 | 9.86 | 0.599 | 4.39E-10 | 1.39E-07 |
| CXCL11 | NM_001161666.1:64 | 8.72 | 0.393 | 1.01E-11 | 1.59E-08 |
| CXCL13 | XM_005553359.1:807 | 5.46 | 0.874 | 2.99E-05 | 0.000628 |
| CXCL2 | XM_001097804.2:1184 | 2.55 | 0.315 | 1.95E-06 | 6.17E-05 |
| CXCL3 | XM_001103518.2:558 | 3.41 | 0.298 | 3.65E-08 | 3.24E-06 |
| CXCL6 | XM_001086084.2:1149 | 2.36 | 0.541 | 0.000783 | 0.0107 |
| CXCL9 | XM_005555011.1:254 | 9.45 | 0.43 | 1.16E-11 | 1.59E-08 |
| CXCR3 | XM_005590317.1:341 | 3.27 | 0.41 | 2.36E-06 | 7.31E-05 |
| CXCR4 | XM_002806456.1:553 | 1.45 | 0.24 | 4.12E-05 | 0.000807 |
| CXCR6 | XM_005564743.1:1361 | 1.71 | 0.474 | 0.0032 | 0.0386 |
| CYBB | XM_005568446.2:2406 | 4.84 | 0.227 | 1.69E-11 | 1.71E-08 |
| DDX58 | XM_005570240.1:356 | 2.72 | 0.316 | 1.00E-06 | 3.61E-05 |
| DEFB1 | NM_001266034.1:115 | -1.27 | 0.271 | 0.000413 | 0.00607 |
| DEFB4A | XM_005541239.1:597 | 3.28 | 0.721 | 0.000542 | 0.00772 |
| DOCK9 | XM_005542122.1:545 | -1.29 | 0.207 | 3.02E-05 | 0.000631 |
| EBI3 | XM_005569954.1:1283 | 1.46 | 0.359 | 0.00131 | 0.0169 |
| EIF2AK2 | XM_005575360.1:388 | 1.75 | 0.233 | 4.43E-06 | 0.000126 |
| EOMES | XM_002808195.1:1136 | 2.5 | 0.476 | 0.000156 | 0.00263 |
| ETS1 | XM_005547319.2:851 | 1.07 | 0.147 | 6.00E-06 | 0.00016 |
| FAS | XM_005564600.1:1760 | 1.68 | 0.2 | 1.36E-06 | 4.55E-05 |
| FASLG | NM_001032806.1:40 | 1.81 | 0.361 | 0.00024 | 0.00384 |
| FCAR | XM_005591262.1:760 | 2.78 | 0.348 | 2.25E-06 | 7.02E-05 |
| FCER1A | XM_005592346.1:1643 | -1.26 | 0.307 | 0.00126 | 0.0162 |
| FCER1G | NM_001077423.1:191 | 2.02 | 0.155 | 7.60E-09 | 1.01E-06 |
| FCGR1A | XM_005576718.1:454 | 4.05 | 0.379 | 8.34E-08 | 5.55E-06 |
| FCGR2A | NM_001032880.1:156 | 1.72 | 0.309 | 9.22E-05 | 0.00164 |
| FCGR3 | XM_001101933.2:476 | 5.43 | 0.446 | 1.76E-08 | 1.85E-06 |
| FEZ1 | NM_001047141.1:330 | -2.09 | 0.333 | 2.79E-05 | 0.000592 |
| GATA3 | NM_001047134.1:415 | -1.57 | 0.256 | 3.45E-05 | 7.00E-04 |
| GBP1 | XM_005586224.1:906 | 6.53 | 0.341 | 6.71E-11 | 3.42E-08 |
| GFI1 | XM_005540874.1:1913 | 1.32 | 0.358 | 0.00268 | 0.0329 |
| GNLY | XM_005557935.1:904 | 4.99 | 0.453 | 5.80E-08 | 4.38E-06 |
| GPR183 | XM_001093763.2:655 | 2.55 | 0.269 | 3.26E-07 | 1.53E-05 |
| GRB2 | XM_005591903.1:1212 | 1.21 | 0.141 | 1.01E-06 | 3.63E-05 |
| GTF3C1 | XM_005540173.2:2022 | -1.18 | 0.13 | 5.68E-07 | 2.35E-05 |
| GZMA | NM_001283167.1:407 | 5.31 | 0.595 | 6.64E-07 | 2.62E-05 |
| GZMB | XM_005542801.1:1863 | 6.79 | 0.485 | 3.24E-09 | 5.29E-07 |
| GZMH | XM_005541196.1:395 | 4.2 | 0.726 | 6.39E-05 | 0.0012 |
| GZMK | XM_001082963.2:300 | 3.7 | 0.435 | 1.12E-06 | 3.89E-05 |
| HAVCR2 | XM_001092271.2:1235 | 2.95 | 0.245 | 1.99E-08 | 1.97E-06 |
| HIF1A | XM_005560994.1:163 | 1.45 | 0.14 | 1.19E-07 | 7.45E-06 |
| HLA-A | XM_001104255.1:180 | 2.22 | 0.172 | 8.32E-09 | 1.05E-06 |
| HLA-B | XM_005546748.1:867 | 2.12 | 0.123 | 2.39E-10 | 9.29E-08 |
| HLA-C | XM_001117605.2:526 | 1.44 | 0.317 | 0.000554 | 0.00783 |
| HLA-DMA | XM_005546908.1:1534 | 2.28 | 0.183 | 1.30E-08 | 1.48E-06 |
| HLA-DMB | XM_001115037.2:2516 | 1.93 | 0.23 | 1.36E-06 | 4.55E-05 |
| HLA-DOB | XM_005577709.1:708 | 1.75 | 0.289 | 4.16E-05 | 0.000812 |
| HLA-DPA1 | XM_005581558.1:1793 | 2.65 | 0.195 | 4.52E-09 | 7.15E-07 |
| HLA-DPB1 | NM_001130426.1:55 | 2.53 | 0.256 | 2.05E-07 | 1.08E-05 |
| HLA-DQA1 | XM_005564912.2:2675 | 2.7 | 0.276 | 2.37E-07 | 1.22E-05 |
| HLA-DQB1 | NM_001044731.1:113 | 2.64 | 0.251 | 1.01E-07 | 6.58E-06 |
| HLA-DRA | XM_001100700.2:975 | 2.96 | 0.237 | 1.31E-08 | 1.48E-06 |
| HLA-DRB1 | NM_001130424.1:487 | 2.66 | 0.216 | 1.58E-08 | 1.70E-06 |
| HSH2D | XM_005586756.1:1085 | 2.24 | 0.418 | 0.000133 | 0.00226 |
| HSPB1 | XM_005578207.1:1153 | -1.46 | 0.26 | 8.77E-05 | 0.00157 |
| ICAM1 | NM_001042644.1:210 | 3.12 | 0.234 | 5.69E-09 | 8.09E-07 |
| ICAM2 | XM_005581114.1:2077 | 1.25 | 0.162 | 3.32E-06 | 9.64E-05 |
| ICAM3 | XM_005596068.1:724 | 1.72 | 0.292 | 5.30E-05 | 0.001 |
| ICOS | NM_001083599.1:760 | 3.05 | 0.333 | 5.08E-07 | 2.14E-05 |
| IDO1 | NM_001032936.1:60 | 9.43 | 0.46 | 2.76E-11 | 2.16E-08 |
| IDO2 | XM_005548811.1:270 | 2 | 0.481 | 0.00113 | 0.0148 |
| IFI16 | XM_005590303.1:1479 | 1.15 | 0.167 | 1.06E-05 | 0.000255 |
| IFI35 | XM_001109557.2:2414 | 2.62 | 0.256 | 1.38E-07 | 8.33E-06 |
| IFI44 | XM_001106168.2:540 | 2.85 | 0.287 | 1.96E-07 | 1.06E-05 |
| IFIH1 | XM_001113810.2:180 | 2.73 | 0.275 | 1.99E-07 | 1.06E-05 |
| IFIT1 | XM_002801104.1:2191 | 3.06 | 0.385 | 2.40E-06 | 7.40E-05 |
| IFIT2 | NM_001032858.1:466 | 3.87 | 0.384 | 1.67E-07 | 9.41E-06 |
| IFIT3 | XM_005572424.1:1339 | 3.33 | 0.36 | 4.40E-07 | 1.92E-05 |
| IFITM1 | XM_005589339.1:1170 | 2.56 | 0.199 | 9.08E-09 | 1.12E-06 |
| IFNAR2 | XM_005589419.1:281 | 1.98 | 0.242 | 1.83E-06 | 5.82E-05 |
| IFNG | XM_005553451.1:1121 | 4.15 | 0.693 | 4.61E-05 | 0.000885 |
| IFNGR2 | NM_001042641.2:145 | 1.91 | 0.167 | 3.75E-08 | 3.27E-06 |
| IKBKE | XM_005545122.1:233 | 1.32 | 0.306 | 0.000828 | 0.0113 |
| IKZF1 | NM_001040675.1:230 | 2.95 | 0.262 | 4.55E-08 | 3.71E-06 |
| IKZF3 | NM_001039950.1:390 | 2.45 | 0.463 | 0.000145 | 0.00245 |
| IL10 | NM_001287688.1:129 | 2.13 | 0.287 | 4.96E-06 | 0.000137 |
| IL10RA | XM_001104337.2:745 | 2.66 | 0.276 | 2.72E-07 | 1.35E-05 |
| IL10RB | XM_002800380.1:156 | 1.18 | 0.171 | 1.06E-05 | 0.000255 |
| IL11RA | NM_001285324.1:514 | -2.17 | 0.348 | 3.10E-05 | 0.000637 |
| IL12RB1 | XM_005593931.1:680 | 2.45 | 0.572 | 0.000895 | 0.012 |
| IL12RB2 | XM_005551523.1:931 | 1.48 | 0.243 | 3.91E-05 | 0.000776 |
| IL15 | NM_001032884.1:178 | 1.65 | 0.193 | 1.09E-06 | 3.86E-05 |
| IL18R1 | XM_005586152.1:1971 | 1.72 | 0.346 | 0.000254 | 0.00398 |
| IL18RAP | XM_005561820.1:826 | 2.59 | 0.549 | 0.000405 | 0.00597 |
| IL1B | XM_005572139.1:3939 | 3.4 | 0.481 | 8.33E-06 | 0.000209 |
| IL1R2 | NM_001040670.1:2367 | -1.45 | 0.391 | 0.0026 | 0.0321 |
| IL1RN | XM_005539655.1:300 | 2.27 | 0.383 | 5.10E-05 | 0.00097 |
| IL21 | XM_005579695.1:1968 | 2.48 | 0.282 | 7.85E-07 | 2.98E-05 |
| IL21R | NM_001171838.1:235 | 4.06 | 0.368 | 5.71E-08 | 4.38E-06 |
| IL27 | XM_005589832.1:340 | 2.23 | 0.352 | 2.59E-05 | 0.000558 |
| IL2RA | XM_005541969.1:751 | 4.2 | 0.567 | 5.13E-06 | 0.00014 |
| IL2RB | XM_001091080.2:375 | 3.71 | 0.363 | 1.40E-07 | 8.33E-06 |
| IL2RG | XM_005553435.1:1550 | 2.93 | 0.358 | 1.71E-06 | 5.52E-05 |
| IL4R | XM_005595004.1:1116 | 1.72 | 0.204 | 1.24E-06 | 4.24E-05 |
| IL6 | NM_001136100.1:1398 | 4.89 | 0.403 | 1.85E-08 | 1.87E-06 |
| IL7R | XM_001100929.2:2035 | 2.91 | 0.408 | 7.61E-06 | 0.000196 |
| IRF1 | XM_005560998.1:576 | 4.37 | 0.276 | 7.24E-10 | 2.15E-07 |
| IRF7 | NM_001047137.1:25 | 5.08 | 0.346 | 1.80E-09 | 3.57E-07 |
| IRF8 | NM_001037092.1:2100 | 4.21 | 0.286 | 1.78E-09 | 3.57E-07 |
| ISG20 | XM_001115072.2:2435 | 3.49 | 0.397 | 7.81E-07 | 2.98E-05 |
| ITGA4 | XM_005540699.1:669 | 2.43 | 0.265 | 4.70E-07 | 2.00E-05 |
| ITGAL | NM_001032821.1:187 | 3.52 | 0.445 | 2.50E-06 | 7.56E-05 |
| ITGAM | NM_001079693.1:1034 | 2.85 | 0.298 | 3.07E-07 | 1.46E-05 |
| ITGAX | NM_001032943.1:89 | 2.4 | 0.314 | 3.68E-06 | 0.000106 |
| ITGB2 | XM_005539939.1:626 | 3.07 | 0.259 | 2.51E-08 | 2.35E-06 |
| JAK2 | NM_001044739.1:364 | 1.8 | 0.195 | 4.65E-07 | 1.99E-05 |
| JAK3 | XM_005577224.2:1183 | 2.99 | 0.445 | 1.44E-05 | 0.000333 |
| KIR3DL1 | XM_002806330.1:1175 | 1.89 | 0.474 | 0.00154 | 0.0197 |
| KLRC1 | XM_005540333.1:1225 | 3.35 | 0.515 | 1.98E-05 | 0.000438 |
| KLRC3 | NM_001042733.1:139 | 3.81 | 0.555 | 1.16E-05 | 0.000273 |
| KLRD1 | NM_001047135.1:1170 | 4.21 | 0.446 | 3.56E-07 | 1.64E-05 |
| KLRF1 | XM_005591267.1:620 | 2.43 | 0.676 | 0.00329 | 0.0396 |
| KLRK1 | XM_005542401.1:734 | 3.81 | 0.348 | 6.23E-08 | 4.57E-06 |
| LAG3 | NM_001032933.1:797 | 2.55 | 0.436 | 5.66E-05 | 0.00106 |
| LAIR1 | NM_001047123.1:655 | 3.17 | 0.312 | 1.48E-07 | 8.69E-06 |
| LAIR2 | XM_005539941.1:1616 | 2.22 | 0.402 | 9.73E-05 | 0.00172 |
| LAMP3 | XM_005560453.2:437 | 2.77 | 0.538 | 0.000183 | 0.00302 |
| LCK | XM_005558492.1:895 | 2.95 | 0.407 | 6.48E-06 | 0.000171 |
| LCP2 | XM_005572307.1:2923 | 3.4 | 0.368 | 4.35E-07 | 1.91E-05 |
| LIF | XM_005595008.1:1551 | 1.7 | 0.389 | 0.000743 | 0.0102 |
| LILRA1 | NM_001261621.2:516 | 1.74 | 0.457 | 0.00214 | 0.0268 |
| LILRA2 | XM_005591740.1:1671 | 3.04 | 0.486 | 2.92E-05 | 0.000615 |
| LILRA3 | XM_005546902.1:1188 | 2.85 | 0.306 | 4.12E-07 | 1.83E-05 |
| LILRA4 | NM_001283284.1:374 | 3.46 | 0.536 | 2.13E-05 | 0.000467 |
| LILRB1 | NM_001284908.1:522 | 2.16 | 0.378 | 7.09E-05 | 0.0013 |
| LILRB3 | XM_005580142.1:2883 | 2.21 | 0.412 | 0.000131 | 0.00225 |
| LILRB4 | NM_001048247.1:214 | 4.26 | 0.585 | 6.12E-06 | 0.000162 |
| LIMK1 | NM_001260949.2:416 | 1.26 | 0.289 | 0.00075 | 0.0103 |
| LITAF | NM_001287632.1:908 | 1.31 | 0.166 | 2.50E-06 | 7.56E-05 |
| LTB | XM_005542938.1:626 | 3.2 | 0.531 | 4.35E-05 | 0.000845 |
| LTF | XM_005553361.1:970 | 4.21 | 0.562 | 4.58E-06 | 0.000129 |
| LY96 | XM_001097884.2:1125 | 2.13 | 0.241 | 7.42E-07 | 2.86E-05 |
| MAP4K1 | XM_001097639.2:645 | 2.83 | 0.247 | 3.58E-08 | 3.23E-06 |
| MBP | NM_001193463.1:758 | -1.21 | 0.196 | 3.25E-05 | 0.000662 |
| MME | XM_005575254.1:1742 | -1.98 | 0.402 | 0.000271 | 0.00418 |
| MMP3 | NM_001284040.1:867 | 3.39 | 0.633 | 0.000131 | 0.00225 |
| MMP9 | XM_005561440.1:2618 | 3.29 | 0.676 | 0.000307 | 0.00465 |
| MR1 | XM_005558115.1:1376 | 1.49 | 0.172 | 8.94E-07 | 3.35E-05 |
| MRC1 | XM_005588318.1:1049 | 1.14 | 0.184 | 3.05E-05 | 0.000632 |
| MS4A1 | NM_001042434.1:441 | 3 | 0.476 | 2.76E-05 | 0.000588 |
| MS4A2 | XM_005541313.1:452 | -1.14 | 0.269 | 0.000979 | 0.013 |
| MSR1 | NM_001283615.1:236 | 2.28 | 0.32 | 7.84E-06 | 0.000199 |
| MX1 | XM_001113553.2:3014 | 4.01 | 0.445 | 5.95E-07 | 2.42E-05 |
| MX2 | XM_005553355.1:281 | 3.94 | 0.354 | 5.02E-08 | 4.01E-06 |
| MYD88 | XM_005589661.1:920 | 2.01 | 0.199 | 1.59E-07 | 9.11E-06 |
| NCF4 | XM_015442082.1:1254 | 2.45 | 0.392 | 3.04E-05 | 0.000632 |
| NFKB1 | XM_001085533.2:555 | 1.01 | 0.122 | 1.51E-06 | 4.96E-05 |
| NFKB2 | XM_005584045.1:1828 | 1.08 | 0.217 | 0.000247 | 0.00392 |
| NLRP3 | XM_005569889.1:719 | 1.67 | 0.346 | 0.000332 | 0.00495 |
| NOD1 | NM_001047149.1:195 | 1.17 | 0.129 | 5.39E-07 | 2.25E-05 |
| NOD2 | XM_005575305.1:499 | 1.48 | 0.27 | 0.000105 | 0.00184 |
| NT5E | XM_005592202.1:494 | -1.07 | 0.209 | 0.000201 | 0.00328 |
| OAS1 | XM_005558381.1:529 | 3.8 | 0.395 | 2.85E-07 | 1.38E-05 |
| OAS2 | XM_001118361.2:1059 | 4.51 | 0.38 | 2.35E-08 | 2.26E-06 |
| OAS3 | XM_001092240.2:156 | 2.47 | 0.23 | 7.90E-08 | 5.45E-06 |
| OASL | XM_005554514.1:1197 | 5.06 | 0.484 | 1.06E-07 | 6.79E-06 |
| PDCD1 | XM_001094235.1:882 | 1.64 | 0.35 | 0.000434 | 0.00628 |
| PDCD1LG2 | XM_005588917.1:524 | 3.3 | 0.345 | 3.00E-07 | 1.44E-05 |
| PIGR | XM_002803170.1:1684 | 1.61 | 0.38 | 0.000989 | 0.0131 |
| PIK3C2G | XM_005567685.1:945 | -1.58 | 0.4 | 0.00165 | 0.0209 |
| PLA2G2A | XM_001118373.2:324 | 6.47 | 0.666 | 2.48E-07 | 1.26E-05 |
| PLA2G6 | XM_001097616.2:438 | -1.46 | 0.345 | 0.000987 | 0.0131 |
| PLAU | XM_005590334.1:128 | 1.24 | 0.271 | 0.000512 | 0.00733 |
| PLAUR | NM_001195749.1:233 | 1.48 | 0.305 | 0.000309 | 0.00466 |
| PLCB1 | XM_005555273.1:1317 | -1.33 | 0.268 | 0.000257 | 0.00401 |
| PML | NM_001040672.1:52 | 1.36 | 0.144 | 3.40E-07 | 1.58E-05 |
| POU2F2 | XM_001109319.2:329 | 2.8 | 0.339 | 1.55E-06 | 5.05E-05 |
| PPARG | XM_001095833.2:363 | -1.07 | 0.277 | 0.00195 | 0.0246 |
| PPP1R12B | XM_002807975.1:2245 | -1.32 | 0.146 | 5.74E-07 | 2.36E-05 |
| PRF1 | NM_001042662.1:796 | 4.6 | 0.409 | 4.49E-08 | 3.71E-06 |
| PRKCB | NM_001136101.1:810 | 2.04 | 0.321 | 2.49E-05 | 0.000538 |
| PRKCD | XM_005589553.1:498 | 1.02 | 0.236 | 0.000851 | 0.0115 |
| PSMB10 | NM_001283192.1:339 | 3.82 | 0.263 | 2.02E-09 | 3.78E-07 |
| PSMB8 | XM_005548517.1:853 | 3.75 | 0.199 | 8.30E-11 | 3.81E-08 |
| PSMB9 | NM_001134298.1:360 | 3.89 | 0.236 | 4.41E-10 | 1.39E-07 |
| PTAFR | NM_001283816.1:1043 | 1.07 | 0.222 | 0.000328 | 0.00492 |
| PTGIR | XM_001092778.2:630 | 1.97 | 0.284 | 1.04E-05 | 0.000252 |
| PTGS2 | XM_005581792.1:3097 | 2.92 | 0.51 | 7.02E-05 | 0.00129 |
| PTK2 | XM_005542580.1:955 | -1.11 | 0.169 | 1.86E-05 | 0.000418 |
| PTPN2 | XM_001098400.2:935 | 1.15 | 0.176 | 1.95E-05 | 0.000435 |
| PTPN22 | XM_005557849.1:212 | 2.41 | 0.369 | 1.88E-05 | 0.00042 |
| PTPN6 | NM_001047150.1:222 | 1.55 | 0.22 | 8.87E-06 | 0.00022 |
| PTPRC | XM_001117618.2:1032 | 3.07 | 0.289 | 8.87E-08 | 5.83E-06 |
| RARRES3 | NM_001077483.1:133 | 6.9 | 0.361 | 6.76E-11 | 3.42E-08 |
| RELB | XM_005548232.2:858 | 1.84 | 0.275 | 1.49E-05 | 0.000345 |
| RORC | XM_005582995.1:465 | -1.48 | 0.294 | 0.000227 | 0.00366 |
| RUNX1 | NM_001285192.1:191 | 1.14 | 0.151 | 4.19E-06 | 0.00012 |
| S100A8 | NM_001261614.1:1598 | 6.8 | 0.872 | 2.98E-06 | 8.80E-05 |
| S100A9 | XM_001083654.2:1670 | 7.31 | 1.05 | 1.04E-05 | 0.000252 |
| SELE | XM_005549884.1:2719 | 2.86 | 0.39 | 5.61E-06 | 0.00015 |
| SELL | XM_002803985.1:555 | 4 | 0.411 | 2.48E-07 | 1.26E-05 |
| SELPLG | NM_001040676.1:635 | 2.18 | 0.42 | 0.000175 | 0.00291 |
| SERPING1 | XM_005548166.1:1134 | 1.46 | 0.156 | 4.01E-07 | 1.79E-05 |
| SH2D1A | XM_001099491.2:702 | 3.77 | 0.395 | 3.10E-07 | 1.47E-05 |
| SIGLEC1 | NM_001195651.1:1020 | 2.84 | 0.194 | 1.84E-09 | 3.57E-07 |
| SLAMF1 | XM_005560599.1:2185 | 2.11 | 0.367 | 6.87E-05 | 0.00127 |
| SLAMF6 | XM_015433780.1:1390 | 2.3 | 0.625 | 0.0028 | 0.0342 |
| SLAMF7 | NM_001130427.1:120 | 5.29 | 0.545 | 2.54E-07 | 1.27E-05 |
| SMAD5 | XM_005553160.1:259 | -1.44 | 0.183 | 2.71E-06 | 8.15E-05 |
| SMPD3 | XM_001111368.2:405 | -1.03 | 0.258 | 0.00156 | 0.0199 |
| SOCS1 | NM_001032828.1:365 | 2.99 | 0.338 | 7.14E-07 | 2.78E-05 |
| SOCS3 | XM_005565623.1:1621 | 3.59 | 0.328 | 6.34E-08 | 4.58E-06 |
| STAT1 | XM_001085792.2:202 | 3.27 | 0.161 | 2.99E-11 | 2.16E-08 |
| STAT2 | XM_005543945.1:2291 | 1.93 | 0.176 | 5.75E-08 | 4.38E-06 |
| STAT5A | XM_005578241.1:812 | 1.51 | 0.177 | 1.10E-06 | 3.86E-05 |
| SYK | XM_001107711.2:2036 | 2.65 | 0.263 | 1.67E-07 | 9.41E-06 |
| TAGAP | NM_001032959.1:58 | 2.17 | 0.365 | 4.90E-05 | 0.000935 |
| TAP1 | XM_005553343.1:545 | 3.14 | 0.235 | 5.76E-09 | 8.09E-07 |
| TAP2 | XM_005592879.1:540 | 3.22 | 0.255 | 1.12E-08 | 1.31E-06 |
| TAPBP | XM_001093060.2:843 | 1.83 | 0.11 | 3.79E-10 | 1.37E-07 |
| TARP | XM_001105753.2:840 | 2.36 | 0.471 | 0.000239 | 0.00384 |
| TBX21 | XM_005592304.1:741 | 4.71 | 0.415 | 4.03E-08 | 3.39E-06 |
| TGFB1 | XM_005580959.1:769 | 1.44 | 0.17 | 1.21E-06 | 4.15E-05 |
| TGFB2 | XM_005551982.1:1536 | -1.56 | 0.276 | 7.78E-05 | 0.00141 |
| TIGIT | XM_005591741.1:2025 | 2.34 | 0.529 | 0.000697 | 0.00968 |
| TLR1 | NM_001032850.1:170 | 2.49 | 0.287 | 9.32E-07 | 3.41E-05 |
| TLR2 | NM_001032879.1:202 | 3.68 | 0.246 | 1.42E-09 | 3.21E-07 |
| TLR3 | NM_001134900.1:345 | 1.2 | 0.254 | 0.000388 | 0.00576 |
| TLR4 | XM_001086302.2:1204 | 3.24 | 0.242 | 5.49E-09 | 8.09E-07 |
| TLR6 | XM_005570089.2:434 | 1.3 | 0.231 | 8.05E-05 | 0.00145 |
| TLR7 | XM_005540653.1:448 | 2.25 | 0.27 | 1.42E-06 | 4.70E-05 |
| TLR8 | XM_002803688.1:1486 | 2.54 | 0.162 | 7.96E-10 | 2.24E-07 |
| TMEM173 | XM_005541789.1:223 | 2.01 | 0.135 | 1.46E-09 | 3.21E-07 |
| TNF | NM_001042642.1:320 | 2.53 | 0.468 | 0.000119 | 0.00206 |
| TNFAIP3 | XM_005567397.1:536 | 1.71 | 0.328 | 0.000168 | 0.00281 |
| TNFAIP6 | XM_005549595.1:528 | 3.58 | 0.253 | 2.79E-09 | 5.05E-07 |
| TNFRSF11A | XM_005545584.1:1990 | 1.08 | 0.304 | 0.00358 | 0.043 |
| TNFRSF13B | XM_005553065.1:581 | 1.77 | 0.472 | 0.00244 | 0.0303 |
| TNFRSF14 | XM_002799538.1:945 | 1.36 | 0.242 | 8.37E-05 | 0.0015 |
| TNFRSF18 | NM_001035529.1:645 | 2.49 | 0.502 | 0.000264 | 0.00411 |
| TNFRSF1B | NM_001284177.1:95 | 1.81 | 0.233 | 3.12E-06 | 9.16E-05 |
| TNFRSF4 | NM_001032917.1:652 | 2.24 | 0.394 | 7.58E-05 | 0.00138 |
| TNFSF13 | XM_005569995.1:662 | 2.08 | 0.203 | 1.39E-07 | 8.33E-06 |
| TNFSF13B | XM_001112861.2:789 | 3.28 | 0.172 | 6.74E-11 | 3.42E-08 |
| TNFSF8 | NM_001287657.1:358 | 1.51 | 0.262 | 6.66E-05 | 0.00124 |
| TRAF1 | NM_001195389.1:825 | 2.35 | 0.357 | 1.75E-05 | 4.00E-04 |
| TRAF3 | XM_005559876.1:1523 | 1.03 | 0.152 | 1.26E-05 | 0.000294 |
| TREM2 | XM_005541787.1:236 | 2.39 | 0.374 | 2.37E-05 | 0.000515 |
| TYROBP | NM_001284968.1:765 | 2.72 | 0.261 | 1.09E-07 | 6.89E-06 |
| VCAM1 | XM_002803112.1:150 | 1.17 | 0.284 | 0.00123 | 0.0159 |
| XCL1 | XM_005582776.1:980 | 5.23 | 0.489 | 8.26E-08 | 5.55E-06 |
| XCR1 | XM_005570039.1:339 | 1.89 | 0.456 | 0.00114 | 0.0149 |
| ZAP70 | XM_005542274.1:2386 | 1.83 | 0.41 | 0.000635 | 0.00885 |
| ZBTB16 | XM_005586643.1:1136 | -2.49 | 0.305 | 1.78E-06 | 5.70E-05 |
| ZNF205 | XM_015457667.1:1451 | -1.16 | 0.215 | 0.000118 | 0.00205 |
