# Supplementary material for: CpG ODN D35 improves the response to abbreviated low-dose pentavalent antimonial treatment in non-human primate model of cutaneous leishmaniasis
Source: PLoS Negl Trop Dis. 2020 Feb 28;14(2):e0008050. doi: 10.1371/journal.pntd.0008050 (PMC7075640; doi:10.1371/journal.pntd.0008050)
Supplement: Attachment — Submitted filename: review 1 for PLOS.docx [file pntd.0008050.s015.docx]

The paper presented by Thacker et al is about the efficacy of one dose CpG ODN D35 with low dose antimonial treatment in L. major infected primates. Their results showed that by using this approach, macaques had smaller lesions and reduced time to re-epithelization but almost no effect on parasite load.

The manuscript is very important in the field of CL treatment especially the data presented in macaques. The main problem in this manuscript is incorporation of too many data which indeed very hard to follow. Especially the graphs are not prepared properly and are very hard to read due to used small letters on graph heading.

**M@M section**

Line 151, "Buffy coats from healthy blood donors" Please mention healthy human blood donor?

Line 163-169: why for CAL‐ 1 cells, the combination of D35+ SbV did not use? Is there specific reason for time variation sometimes it is 24 hr for PBMC and 18hr for CAL‐ 1 cells ?

Lines 197-200: please clarify this sentence: "CpG ODN injections were performed on alternating sides of the thorax and at least 2 inches away from a previous inoculation site"

Line231: "promastigotes on the forehead at three separate sites (1 on the right and 2 on the left)"what is the reason? Why the total parasites did not injected at one point?

Line 229: Please define the treatment groups as groups 1 , 2, 3, 4 similar to the text 458-459.

Line 238: please define DPI

**Results section:**

Lines 305 -332: why the authors did not use macaques instead of human PBMC? What was the limitation? Please harmonize the term D35 or D ODN or ODN….it is better to use D35 throughout the manuscript. Define MX-1 in Fig 1D and E.

Line 307: please change to L. major

Line 324: what about IFN-g in Fig 1C?

Line 324: Fig 1D needs more explanation.

Line 329-332: Please make it clear which D35 did you use for whole study in primate, ultrapure or Research grade?

Fig 2 A-D: in some part the control macaque is missing (purple line), it is really hard to understand this figure very well. In this section D35 is used every 3 weeks for 6 times? Please clarify.

Fig 2 G-L: what is the orange line? G11 shown with purple line which is only appear on first injection period! Very hard to understand.

Line 353: What is the reason to have considerable expression of interferon stimulated genes (ISGs), *MX-1, OAS-1, CXCL-10*, and *IRF7*, in contralateral side of the torso?

There is no graph to show the ISGs in Figure 2. In Fig 2-C there is no control at week 18.

In Fig 2 B in contralateral side, at week 12 and 18 there is confused up and down expression for OAS1, what are the reasons?

In Fig 2 E and F, for primate C07, there is upper level of IL-6 and IL-8 in contralateral side. Altogether this graph shows lots of variation and there is no concrete conclusion. This variation is within 3 macaques. Instead of graphs, could be easier to show them in a table (mean+/- SD) with their p value.

Line 387: this section is on human PBMC and the action of Sb and D35 at in vitro. In order not to back and forth from human to macaques, highly suggest describing everything in human first and then continued with in vitro and in vivo experiments in macaques.

Line 398, The data for ISG is missing?

Why the n is different for gene and protein evaluation? In Fig 3 mentioned untreated marked as circle blue and D35 as red cube. Please define the meaning of each clearly. In Fig 3A and B, why there is different view in the case of IL-6? There is no IL-6 production at protein level.

Line 418: In section "In vivo effects of SbV on D35 treatment" the authors used different gene expression for macaques (MX-1, CXCL-10 and IRF-7) and in the case of human IFN-a, CXCL10, IFN-g and IL-6, why is that so?

Line 453: please indicate as group 1 (saline controls), group 2( SbV 5 mg/kg (SbV

lo), group 3 D35 1 mg/kg (D35), and group 4 (Sb lo plus D35). The Sup Fig 3 A is confusing and there are different flashes below the line with no meaning.

Line 492: the Fig 5, J is missing, should be Fig5J, L-Q.

Fig 6 D, please harmonize the y axis and all should be fixed at 10.

My major critic is in Supplementary Fig 6. This Figure is very important and should be among the essential figure not in supplementary. If there is an improvement in the lesion size, why there is no significant difference between Sb+D35 and saline or other groups. Even at D71 almost no parasite DNA was seen on saline and D35 but existed on Sb and Sb+D35. If the minicircle DNA is very stable why there are huge amount of differences between D22 and D71. It is highly suggest repeating the parasite detection with different set of primers such as RV1 and RV1 and check the differences on D11, D22 and D71.

**Discussion section:**

Lines 568-570: need revision

Lines 668-680: it is better to use another set of primers for parasite burden and then conclude this section. This is hard to believe unless prove it by another approach

The rest of discussion is clear and reads well. It seems that different sections are written by different co-authors so some parts are written very well and some parts are very hard to understand. Therefore highly suggest improving and clarifying the result and M&M sections for reading.
